# Risk factors and assessment methods for screening childhood-onset fluency disorders: a scoping review

**DOI:** 10.1590/2317-1782/e20250336en

**Published:** 2026-07-06

**Authors:** Mayra Maria Oliveira de Lima, Heloísa de Oliveira dos Anjos, Thaíse Sara Costa Dias, Débora Vasconcelos Correia, Cristiane Moço Canhetti de Oliveira

**Affiliations:** 1 Programa de Pós-graduação em Ciências da Saúde e Comunicação Humana, Universidade Estadual Paulista “Júlio de Mesquita Filho – UNESP - Marília (SP), Brasil.; 2 Programa de Pós-graduação em Linguística, Universidade Federal da Paraíba – UFPB - João Pessoa (PB), Brasil.

**Keywords:** Risk Factors, Stuttering, Childhood-Onset Fluency Disorder, Child Development, Child Health, Speech-Language Pathology, Scoping Review

## Abstract

**Purpose:**

To map the state of the art regarding risk factors and assessment methods employed in the screening of fluency disorders in preschool children.

**Research strategies:**

A scoping review was conducted in accordance with the Joanna Briggs Institute guidelines and the PRISMA-ScR checklist. The guiding question was: What are the risk factors and assessment methods used in the screening of fluency disorders (stuttering and cluttering) in preschoolers, as reported in the international literature? The PCC strategy guided the search, using MeSH descriptors and free terms across the Lilacs, PubMed/Medline, Scopus, and ScienceDirect databases, from 2020 to 2025, with no language restrictions.

**Selection criteria:**

Original full-text articles addressing at least one research question were included. Exclusion criteria comprised non-scientific or secondary publications, studies without abstracts, those outside the conceptual or population scope, and those involving co-occurring disorders.

**Data analysis:**

Data were analyzed descriptively and synthesized narratively.

**Results:**

A total of 2,091 publications were identified; after duplicate removal and eligibility screening, 11 articles were included. No studies on risk factors for cluttering were found. For stuttering, 14 predictive risk factors were mapped, related to body functions and structures as well as contextual factors. Only two standardized screening tools were identified, both developed in Brazil: the Developmental Stuttering Risk Protocol and the Developmental Stuttering Screening Instrument.

**Conclusion:**

Evidence on risk factors for cluttering in preschoolers is lacking. For stuttering, biological and contextual factors emerged as key predictors. Most available assessment methods are restricted to use by speech-language pathologists, highlighting gaps and opportunities for future research.

## INTRODUCTION

The most well-known fluency disorders with onset in childhood are stuttering and cluttering. Involuntary disfluencies primarily characterize stuttering, and these primary symptoms compromise the usual and automatic flow of speech^(1)^. In addition, secondary behaviors may be present, such as the substitution or avoidance of words, in an attempt to minimize fluency difficulties^(2)^. Cluttering is characterized by a speech rate perceived as fast and/or irregular, accompanied by at least one of the following symptoms: increased occurrence of disfluencies, most of which are not typical of stuttering; coarticulation between sounds; and pauses in atypical positions^(3)^.

Both disorders affect speech timing patterns, have a strong genetic component with heritability characteristics, and show a predominance of persistence in males^(4-6)^. In addition, stuttering and cluttering can occur in isolation or coexist^(7)^. Despite the prevalence of childhood-onset fluency disorders affecting approximately 5% of the preschool population^(8)^ in cases of stuttering, and up to 1.2% in adolescents in cases of cluttering^(9)^, their diagnosis is still challenging and often delayed.

For this reason, experts advise that the fundamental initial step in assessing childhood fluency is to carefully investigate risk factors for the development of these disorders^(10,11)^. The more risk factors a child has, the greater the chance that the child will need specialized monitoring^(12-14)^. Speech-language pathology clinical guidelines recommend assessing risk factors for childhood-onset fluency disorders in children under six years of age as a basis for decision-making^(15,16)^.

Risk factors are predictive markers of future health problems, important for guiding diagnostic and therapeutic decisions^(17)^. In stuttering, among the various risk factors identified in the literature, we can mention: male sex, caregiver complaints, family history, age of onset of the disorder, and duration of the complaint, among others^(11-14,18-21)^. In cluttering, some risk factors are similar to those in stuttering, such as sex and family history, but it differs in considering disorganized language planning and the absence or low perception of communicative difficulty^(22,23)^.

However, despite both conditions being fluency disorders that begin in childhood, no formal instruments are available to screen for them together. As stuttering is the most studied fluency disorder, there is a noticeable effort by the scientific community to screen for it, as exemplified by the instruments: Protocolo de Risco para a Gagueira do Desenvolvimento (PRGD - Developmental Stuttering Risk Protocol)^(24)^ and the Instrumento de Rastreio para a Gagueira do Desenvolvimento (IRGD - Developmental Stuttering Screening Instrument)^(25)^. However, this is not observed with regard to cluttering, as there are no instruments to identify it early in the preschool population.

The assessment methods predominantly used in screening risk factors for stuttering and cluttering advocate specialized clinical assessment, with instruments for the exclusive use of speech-language pathologists, such as the PRGD^(24)^, for preschoolers and school-aged children, and the Cluttering Inventory-Revised (PCI-r)^(26)^, for school-aged children and adolescents, which makes multiprofessional screening actions unfeasible. Given that the Brazilian public health system establishes screening policies for communicative disorders in early childhood^(27-29)^, it remains unclear which risk factors should be considered for screening preschoolers for stuttering and cluttering, and which assessment methods would be most appropriate for such an investigation.

Given this scenario, it is necessary to identify and synthesize the available research on risk factors and assessment methods for screening fluency disorders with onset in childhood. This need aims to provide an overview of the topic, identify gaps and trends, and identify common terms, which aligns with a scoping literature review approach. Thus, we encounter the following research question: What are the risk factors and screening assessment methods used to identify fluency disorders (stuttering and cluttering) in preschoolers in the international literature? The objective of this scoping review was to map the state of the art regarding risk factors and assessment methods used in screening for fluency disorders in preschoolers.

## METHOD

### Protocol and register

We developed this scoping review (SR) of the literature according to the methodology proposed by the Joanna Briggs Institute (JBI), in line with the recommendations of the Reporting Items for Preferred Systematic Reviews and Meta-Analyses extension for Scoping Reviews (PRISMA-ScR) guide. One indication for conducting an SR is when the aim is to identify the main characteristics or factors related to a concept^(30)^, as in the present research. The protocol of this SR was registered on the Open Science Framework platform^(31)^.

### Research questions

To guide the formulation of the guiding question for this review, the study adopted the Population, Concept, and Context (PCC) strategy. Thus, the following was defined: population: preschool children with fluency disorders (stuttering and/or cluttering), aged between 2 and 5 years and 11 months; concept: risk factors and assessment/screening; and context: international scientific literature. Therefore, the following primary research question (RQ) was obtained: What are the risk factors and assessment methods used in the screening of fluency disorders in preschool children in the international literature? The secondary research questions chosen were: What risk factors were identified? (RQ1); What assessment methods were used? (RQ2); What limitations were described in the studies? (RQ3); What suggestions were mentioned by the authors for new research in the area? (RQ4).

### Eligibility criteria

Publications that did not qualify as scientific articles were excluded, such as: letters to the editor, book chapters, abstracts from scientific event proceedings, opinion articles; secondary articles (literature reviews); articles without abstracts; articles outside the conceptual and population scope of interest; or with co-occurrence of other disorders, such as: intellectual disability, neuromotor dysfunction, Attention Deficit Hyperactivity Disorder (ADHD), Autism Spectrum Disorder (ASD), and trisomy 21 (Down syndrome). There was no exclusion based on language, ethnicity, race, sex, gender, or geographic location.

Therefore, the selection included scientific articles available in full, published between 2020 and 2025, and that answered at least one research question. Technical and empirical articles that proposed to discuss theoretical models and/or clinical guidelines for assessing risk factors for the population of interest were also included.

The age range of children up to 5:11 years was delimited as it corresponds to the critical period for the emergence of fluency disorders with onset in childhood^(32)^. The temporal scope of the publications was limited to the last five years to ensure the information collected was based on the most recent scientific evidence in the literature. Only articles available in full were selected, enabling data collection for the SR and achieving the proposed objective. It was decided to include technical and empirical articles to expand the data on risk factors and to understand the guidelines for assessing these factors. The presence of other disorders co-occurring with fluency disorders was excluded so that the risk factors and assessment methods identified would be related exclusively to the population of interest, i.e., preschool children diagnosed with or at risk for stuttering and cluttering.

### Databases

The databases used were Lilacs (Literatura Latino-Americana e do Caribe em Ciências da Saúde), Medical Literature Analysis and Retrieval System (PubMed/Medline), Scopus, and ScienceDirect. The search was conducted on June 30, 2025. The ResearchGate platform was used to actively search for the authors of selected articles that were not available in full after the screening stage, to preserve the sample of studies screened and selected for full-text reading and analysis in the eligibility stage. This active search took place on July 31, 2025.

### Search strategies

Initially, the most frequently used and cited words in diagnostic manuals and clinical guidelines in the area were identified to refer to the concepts and populations of interest in this SR. Therefore, the electronic search strategy was defined as combinations of MESH (Medical Subject Headings) descriptors and free terms, in English, considered relevant to the research, and adapted to the search standards established in each database, as shown in Chart 1.

**Chart 1:** Presentation of search strategies in databases

**Databases**	**Search strategies**
Lilacs, Pubmed/Medline and ScienceDirect (via Portal CAPES)	((child OR "early childhood" OR preschool) AND (stuttering OR stammering OR cluttering OR "fluency disorder") AND ("risk factor" OR assessment OR screening))
Scopus (via Portal CAPES)	((child OR "early childhood" OR preschool OR children) AND (stuttering OR stammering OR cluttering OR "fluency disorder") AND ("risk factor*" OR "predisposing factor*" OR "contributing factor*") AND (assessment OR screen*))

Source: Prepared by the authors, 2025

The validation of the search strategy followed the steps recommended by the Joanna Briggs Institute and the PRISMA-ScR checklist: 1) preliminary search for articles, to verify the sensitivity and specificity of the strategy; 2) reading some abstracts of articles with potential for inclusion in the review; and 3) validation by a speech-language pathologist specializing in Fluency, not involved in the research team. In this way, it was possible to identify the need for adjustments to the search term combination, obtain suggestions for improvement, and conclude the validation of the chosen search strategy.

### Selection of studies

**Figure 1:**
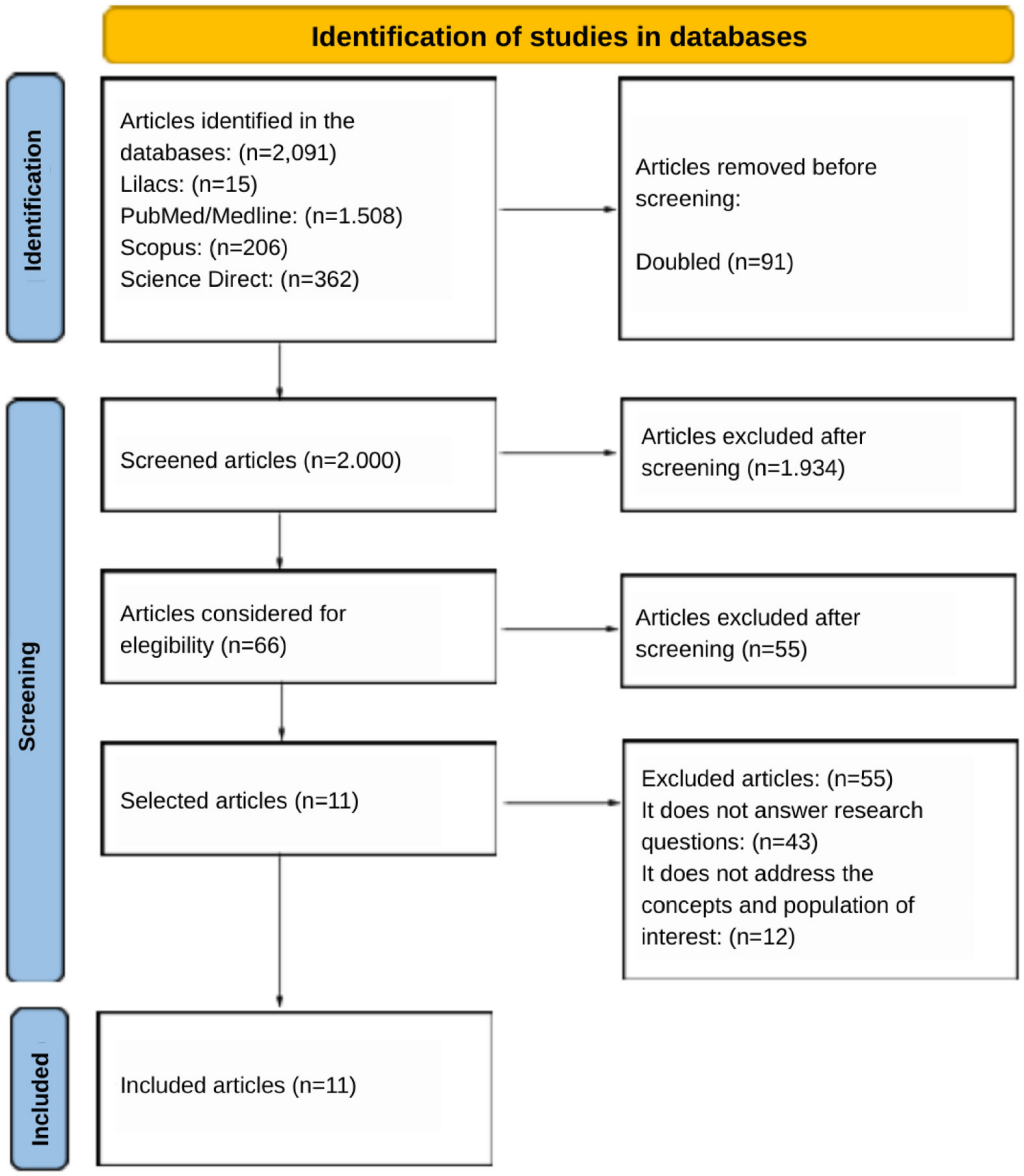
Flowchart of the article selection strategy

Source: Prepared by the authors, 2025.

The selection of articles took place in four stages (Figure 1): identification, screening, eligibility, and inclusion. The Rayyan software was used for the first three stages^(33)^. The main investigator coordinated the use of the software and was responsible for training two independent reviewers, both doctoral speech-language pathologists with expertise in fluency. The reviewers participated in the screening, eligibility, and inclusion stages. The reviewers were trained during the screening stage on 42 articles. After the training, the SR began. Decisions on any disagreements were resolved by consensus to ensure the quality of decision-making and included the participation of a third reviewer when necessary.

The first stage (identification) was carried out by the main investigator, including controlling duplicate publications and quantitatively identifying the articles to be analyzed using database searches. The second stage (screening) was carried out by applying the exclusion criteria, by reading the title, abstract, and keywords. The third stage (eligibility) involved analyzing the inclusion criteria by reading the full article.

### Data extraction, analysis and synthesis

In the fourth stage, inclusion, we extracted the responses to the research questions from each selected article. A Microsoft Excel spreadsheet form, previously developed and calibrated by the team, was used to extract data. The data were collected independently and blindly by the trained reviewers and subsequently consolidated by the principal investigator. The variables for which data were collected in each stage of the review process were:

1. *Identification*: total number of publications identified; total number of publications identified by database; total number of duplicate publications; total number of publications selected by database (after eliminating duplicates).
2. *Screening*: type of publication; possibility of addressing the concepts and population of interest; presence of comorbidity(ies) in the population of interest.
3. *Eligibility and Inclusion*: availability of the full article; risk factors; evaluation methods; limitations described; suggestions made.
4. *Characterization of included articles*: title; year of publication; database; journal; author(s); objective; language; country; target audience; age; sample size (if applicable); type of research (according to the level of scientific evidence).

Specifically regarding the risk factors and assessment methods variables extracted in the eligibility and inclusion stages, a terminological standardization process was necessary for the analysis and synthesis of the results. The terms used to designate the risk factors and assessment methods used in the included articles were grouped by the team based on the following criteria: 1) similarity of meaning, and; 2) selection of preferred terms, favoring the most frequent and current ones in the literature. Thus, it was possible to develop a controlled vocabulary of the review in a table and use the standardized terms in the synthesis of the results.

Although a critical evaluation of the included articles was not carried out, in line with the objectives of scoping reviews, all articles were characterized in terms of their level of evidence using the Agency for Healthcare Research and Quality (AHRQ)^(34)^ to explain their methodological quality. The results were analyzed descriptively, synthesized narratively, and presented in figures and tables. The implications for clinical practice and future research were also discussed.

## RESULTS

### Selection of studies

Through electronic database searches, it was possible to retrieve 2,091 publications, with 15 in Lilacs, 1,508 in PubMed/Medline, 206 in Scopus, and 362 in ScienceDirect. 91 duplicate publications were excluded. 2,000 publications were screened by titles, abstracts, and keywords. After applying the exclusion criteria, 1,934 publications were excluded. 66 articles were selected for full-text reading; of these, after applying the inclusion criteria, 55 articles were excluded. At the end of the eligibility stage, 11 articles were included in the SR for analysis, qualitative synthesis, and description of the results (Figure 1).

### Characterization of included articles

Five articles were obtained from the Scopus database, four from Lilacs, one from PubMed/Medline, and one from ScienceDirect. Six studies were Brazilian^(12,13,18,20,25,35)^, two were American^(14,21)^, two were Dutch^(11,36)^, and one was Japanese^(19)^, with publication years ranging from 2021 to 2025. Only three articles were classified as theoretical studies (levels VI and VII of evidence)^(11,25,36)^; the others^(12-14,18-21,35)^ were distributed from level III to level VI of the Agency for Healthcare Research and Quality (AHRQ) classification of scientific evidence^(34)^. The study population ranged in age from 2 to 12 years, both sexes, with sample sizes ranging from 30 to 2,055 participants. All included articles considered children who stutter as the population of interest. No studies were found that focused on screening and/or risk assessment for cluttering in preschoolers (Table 1).

**Table 1:** Characterization of the articles included in the scoping review

Authors (Year)	Database	Journal	Objective	Level of evidence	Fluency disorder screened	Sample	Age range (years)	Language	Use of any screening instrument?
						(if applicable)		(Country)	
Lima et al. (2021a)	Lilacs	Cefac	To develop a screening tool for identifying the risk of developmental stuttering in preschool children	Observational, analytical, and cross-sectional	Stuttering	NA	2-5	Portuguese (Brazil)	Yes
				Level VI					
Lima et al (2021b)	Lilacs	Cefac			Stuttering	30	2-5	Portuguese (Brazil)	Yes
			To check the sensitivity and accuracy measures of the IRGD	Observational, analytical, and cross-sectional					
				Level VI					
Pinto et al. (2021)	Lilacs	Audiology Communications Research	To test the variable of familial heredity for GCD as a predictor of direct effect on speech fluency outcome in children		Stuttering	200	2-12	Portuguese (Brazil)	Yes
				Case-control study					
				Level IV					
Walsh et al. (2021)	Scopus	Journal of Speech, Language and Hearing Research	To investigate how epidemiological and clinical factors collectively predict whether a preschool-aged child who stutters will persist or recover, and to provide guidance on how clinicians can use these factors to assess a child's risk of stuttering and its persistence	Cohort	Stuttering	52	3-5	English (USA)	Yes
				Level IV					
Ávila et al (2022)	Lilacs	CoDAS	To design a clinical treatment trial – in three modalities – that verifies whether the tested treatments present indicators that allow gathering information for the continuation of their application, establishing an effective and safe benefit-risk ratio.	Non-randomized clinical trial	Stuttering	93	2-12	Portuguese (Brazil)	Yes
				Level III					
Costa et al. (2022)	Scopus	Int. J. Environ. Res. Public Health	To correlate the speech fluency characteristics of children whose parents reported stuttering behaviors with risk factors for persistent stuttering	Observational and prospective	Stuttering	417	2-11	Portuguese (Brazil)	No
				Level VI					
Singer et al (2022)	Scopus	Journal of Speech, Language, and Hearing Research	To assess whether the presence of an increasing number of predictive factors increases a child's risk of developing persistent stuttering	Documentary and retrospective	Stuttering	67	3-5	English	Yes
				Level VI				(USA)	
			To address all the steps a speech-language pathologist should follow, according to the revised Diretrizes de Gagueira (Stuttering Guidelines) (NVLF, 2020), with a young child who stutters, focusing on the assessment of risk factors and the implementation of monitoring						
								
Tonnis, Ormondt (2022)	Scopus	Stem-Spraak-		Narrative and descriptive	Stuttering	NA	< 6	Dutch	No
		En taalpathologie		Level VII					
								(Netherlands)	
Costa et al. (2023)	PubMed/Medline	CoDAS	To investigate two independent variables considered as possible predictors of cumulative risk for persistent stuttering: family perception of stuttering and the number of speech disruptions	Observational, cross-sectional, and retrospective study	Stuttering	452	3-11	Portuguese (Brazil)	Yes
				Level VI					
Franken et al. (2024)	Science Direct	Journal of Fluency Disorders	To update and expand the Erasmus clinical model, incorporating recent evidence to explain the onset, development, and possible outcomes of stuttering in children	Theoretical narrative study proposing a clinical model	Stuttering	NA	Preschool	English	No
				Level VI				(Netherlands)	
Sakai et al (2025)	Scopus	Folia Phoniatrica et Logopaedica	To investigate the prevalence, cumulative incidence, and risk factors of stuttering in early childhood in Japan	Observational, cross-sectional, and epidemiological	Stuttering	2.055	3		
				Level VI				English	
								(Japan)	Yes

**Caption:** NA = Not applicable. Source: Prepared by the authors, 2025

### Risk factors for stuttering in preschoolers

After standardizing the terms used in the articles to designate risk factors, 27 risk factors for stuttering in the preschool population were identified. The ten most frequently cited risk factors were: positive family history of stuttering (with or without recovery) (n=10)^(11-14,18,19,21,25,35,36)^; high frequency of disfluencies typical of stuttering (n=8)^(12-14,19-21,25,35)^; duration of stuttering (n=8)^(11-14,18,21,25,35)^; negative reaction of the child to their own speech (n=7)^(11-13,18,25,35,36)^; age of the child at the onset of stuttering (n=6)^(11,13,14,19,21,35)^; male sex (n=6)^(11,13,14,19,21,35)^; speech tension (effort to speak) (n=6)^(12,13,18,19,25,35)^; negative social reaction to the child's speech (extended family, school and others) (n=6)^(12,13,18,25,35,36)^; worse performance on standardized articulatory/phonological assessment (n=6)^(11,14,18,21,35,36)^; and complaint of stuttering (n=5)^(12,18-20,25)^ (Table 2).

**Table 2:** Matrix of results from the scope review

Authors (Year)	RQ1		RQ2	RQ3	RQ4
	Risk factors identified	Most predictive risk factors (outcomes)	Evaluation methods	Limitations	Suggestions
	16			Limitations to the instrument development process itself, as it is in the initial phase of the overall validation process. Evidence of content validity is essential, but not sufficient.	The continuation of psychometric analyses through the use of new accuracy measures
Lima et al. (2021a)	(RF 1, RF 2, RF 3, RF 4, RF 7, RF 8, RF 10, RF 11, RF 12, RF 13, RF 14, RF 15, RF 19, RF 20, RF 21, RF 22)	NSA	IRGD		
			
Lima et al. (2021b)	16	4		The reduced sample size was due to the short data collection period attributed to the global health crisis, and there was no "gold standard" assessment in the field for diagnosing children in the at-risk group	(1) increasing the sample size; (2) comparing at-risk children with clinical diagnosis; (3) applying the instrument by more professionals in order to obtain other psychometric validation analyses; (4) comparing the application in digital format.
	(RF 1, RF 2, RF 3, RF 4, RF 7, RF 8, RF 10, RF 11, RF 12, RF 13, RF 14, RF 15, RF 19, RF 20, RF 21, RF 22)	(RF 8, RF 10, RF 12, RF 14)	IRGD		
	17	3			
Pinto et al. (2021)	(RF 1, RF 3, RF 4, RF 7, RF 8, RF 9, RF 10, RF 11, RF 12, RF 13, RF 14, RF 15, RF 16, RF 17, RF 18, RF 23, RF 24)	(RF 2, RF 5, RF 10)	PRGD	It doesn't mention	It doesn't mention
					
Walsh et al. (2021)					
				
	6	3	Observational methods;	(1) Include a larger sample of children with stuttering at different ages and incorporate additional risk measures	(1) Create an online calculator in which physicians can enter the child's age, relevant epidemiological factors, and other scores collected during an assessment to determine the collective risk of persistent stuttering in each child
	(RF 1, RF 2, RF 3, RF 5, RF 6, RF 9)	(RF 1, RF 2, RF 9)			
			Complementary methods: Speech/language assessment		
Ávila et al. (2022)				(2) Effect of sample size (non-homogeneous groups regarding the number of participants); absence of randomization; control only of the variable % of stuttered syllables, without evaluating other changes related to stuttering (seasonal variability, speech outside the clinic, interactive skills, emotional and environmental profile, among others); (4) It did not contemplate long-term follow-up of children, preventing the analysis of permanent outcome of the clinical picture.	
			
	19	NA	PRGD		It doesn't mention
	(RF 1, RF 2, RF 3, RF 4, RF 5, RF 6, RF 7, RF 8, RF 9, RF 11, RF 12, RF 13, RF 14, RF 15, RF 16, RF 17, RF 18, RF 23, RF 24)		Observational methods;		
			
			Complementary methods: SSI-3		
	7	4	Self-report methods;	(1) Children without stuttering behaviors (NCSC) did not have a family history of stuttering. Perhaps for this reason the study did not identify the variable "family history" as a predictor for stuttering;	Authors suggest including different age ranges in order to analyze the risk factor for late onset of stuttering behaviors.
Costa et al. (2022)	(RF 1, RF 3, RF 4, RF 5, RF 6, RF 7, RF 8)	(RF 3, RF 7, RF 8, RF 10)		(2) Most of the children in this study were younger (the average age was 6.5 years in both groups). Perhaps for this reason it was not possible to verify whether the risk factors "complaint of stuttering for more than 12 months" and "complaint of stuttering before the age of 5" would be predictors for stuttering;	
			Observation methods;	(3) The results of this study were derived from a single institution;	
				(4) The speech analysis was based exclusively on the first 200 fluent syllables and did not use other speech samples, such as oral reading, naming of isolated words, and longer speech samples.	
Singer et al. (2022)					(1) Include data on the child's temperament and reaction to stuttering to explore the negative consequences related to stuttering (beyond persistence) more comprehensively;
			Self-report based methods;	(1) Data availability; it did not include the temperament/emotion domain;	(2) Include weighted DTGs;
	10	5			(3) Include performance on a pseudoword repetition task;
	(RF 1, RF 2, RF 3, RF 5, RF 6, RF 9, RF 16, RF 18, RF 25)	(RF 3, RF 5, RF 9, RF 16, RF 25)	Observational methods;	(2) The measures used were not identical in all cases.	(4) Include the child's self-report of whether or not they are still stuttering;
				
			Complementary methods: TOCS, SSI-3		
Tonnis and Ormondt (2022)	9	4	NSA	It doesn't mention	It doesn't mention
	(RF 1, RF 3, RF 4, RF 5, RF 6, RF 9, RF 16, RF 17, RF 26)	(RF 1, RF 3, RF 5, RF 6).			
				(1) Analysis of the participants' family history;	
				(2) Differences in the percentages and profiles of breakups in groups of boys and girls;	
Costa et al. (2023)	2	2	Self-report based methods;	3) Time of onset of symptoms and considerations about family attitudes.	It mentions that the limitations will be addressed in future studies
	(RF 2, RF 10)	(RF 2, RF 10)			
			Observation methods.		
					
Franken et al.	5	NA	NA	(1) The model is based on theoretical synthesis and narrative literature review, therefore it has no direct empirical validation.	Empirical validation of the model and improvement of the severity and impact scales for stuttering
-2024	(RF 1, RF 4, RF 8, RF 9, RF 27)			(2) It depends on the integration of multiple risk factors (genetic, neurological, linguistic, emotional, social), but the causal relationship between these factors is not yet fully proven;	
				(3) The model is more useful as a clinical and communication tool with families and patients than as high-level scientific evidence.	
			Self-report methods;	(1) Overestimation of incidence and prevalence	It doesn't mention
Sakai et al. (2025)	7	3	Observation methods;	(2) Reduction of estimated incidence and prevalence due to some parents' avoidance of confirming their children's stuttering because of the strong social stigma associated with the questionnaire	
	(RF 1, RF 2, RF 5, RF 6, RF 7, RF 10, RF 17)	(RF 1, RF 10, RF 17)		(3) Based on parental reports, without direct clinical confirmation	
				(4) Possibility of recall bias in parents' responses	
				(5) As children over four years old are not included in the sample of this research, it is not possible to capture all cases of early-onset stuttering in childhood.	

**Caption:** RQ1 = Secondary Research Question 1; RQ2 = Secondary Research Question 2; RQ3 = Secondary Research Question 3; RQ4 = Secondary Research Question 4; RF 1 = Positive family history of stuttering (with or without recovery); RF 2 = High frequency of disfluencies typical of stuttering; RF 3 = Duration of stuttering; RF 4 = Negative reaction of the child towards their own speech; RF 5 = Age of the child at the onset of stuttering; RF 6 = Male sex; RF 7 = Speech tension (effort to speak); RF = – Negative social reaction towards the child's speech (extended family, school, and others); RF 9 = Worse performance on standardized articulatory/phonological assessment; RF 10 = Complaint of stuttering; RF 11 = Pre-, peri-, and postnatal complications; RF 12 = Impairment in speech intelligibility; RF 13 = Pneumophonoarticulatory incoordination; RF 14 = Presence of concomitant physical symptoms (body and/or facial movements); RF 15 = Altered speech rate; RF 16 = Below-average expressive language development; RF 17 = Presence of comorbidities; RF 18 = Impairment in language comprehension skills; RF 19 = Presence of allergies (atopic dermatitis, food allergy, asthma, and rhinosinusitis); RF = – Presence of sleep disturbances (insomnia, snoring, bruxism, nocturnal restlessness); RF 21 = Presence of other disfluencies (repetition of non-monosyllabic words); RF 22 = Demonstration of stuttering awareness (the child realizes they stutter); RF 23 = Type of persistent stuttering onset; RF 24 = Stressful components of quality of life; RF 25 = Severity of stuttering; RF 26 = Above-average language development; RF 27 = Neuroanatomical and functional alterations; IRGD = Instrumento Risco para a Gagueira do Desenvolvimento (Developmental Stuttering Risk Instrument); PRGD = Protocolo de Risco para a Gagueira do Desenvolvimento (Developmental Stuttering Risk Protocol); TOCS = Test of Childhood Stuttering; SSI-3 = Stuttering Severity Instrument; NA = Not applicable. Source: Prepared by the authors, 2025

**Figure 2:**
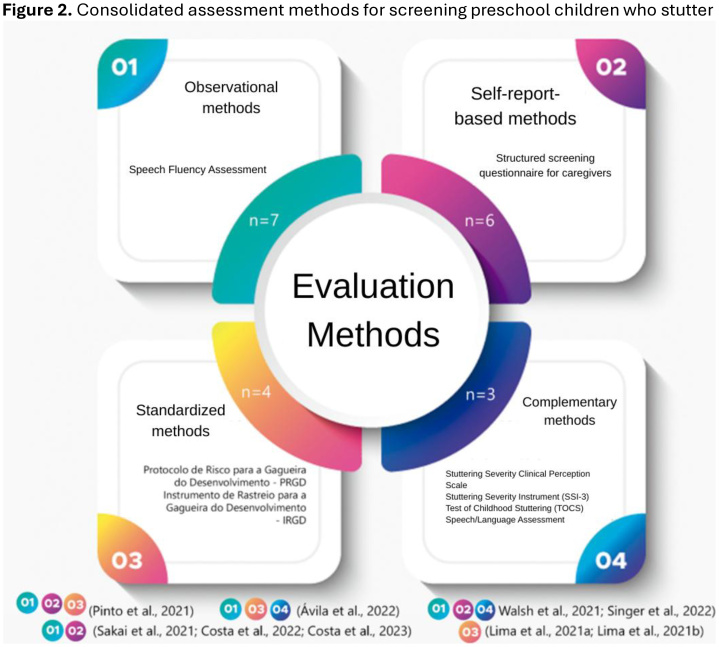
Consolidated assessment methods for screening preschool children who stutter

Source: Prepared by the authors, 2025

Of the eleven articles included, eight investigated which risk factors were most predictive of stuttering. Thus, of the 27 risk factors identified, 14 were most predictive of stuttering. The following seven risk factors were cited as being most predictive of stuttering: complaint of stuttering (n=5)^(12,13,18-20)^; positive family history of stuttering (with or without recovery) (n=3)^(11,19,21)^; child's age at onset of stuttering (n=3)^(11,14,18)^; duration of stuttering (n=3)^(11,13,14)^; high frequency of typical stuttering disfluencies (n=3)^(18,20,21)^; negative social reaction to the child's speech (extended family, school and others) (n=2)^(12,13)^; and poorer performance on standardized articulatory/phonological assessment (n=2)^(14,21)^ (Figure 2).

### Assessment methods used to screen for stuttering in preschoolers

**Figure 3:**
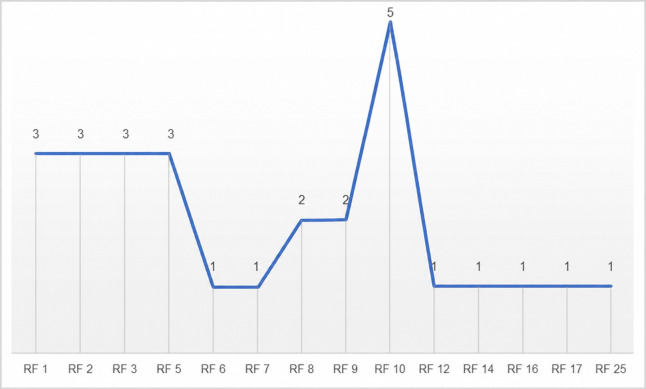
Frequency of the most predictive risk factors for persistent stuttering

After standardizing the terms used in the articles to designate the assessment methods used in stuttering screening in preschoolers, four groups of methods were observed, namely: observational methods (n=7)^(13,14,18-21,35)^, based on the procedures necessary for assessing speech fluency; self-report-based methods (n=6)^(13,14,18-21)^, using a structured screening questionnaire for caregivers; standardized methods for stuttering screening (n=4)^(12,18,25,35)^, based on the use of formal instruments for this purpose; and complementary methods (n=3)^(14,21,35)^, based on the use of perceptual-clinical severity scales of stuttering (n=1)^(21)^, the application of the Stuttering Severity Instrument-3 (SSI-3) (n=2)^(14,35)^, the Test of Childhood Stuttering (TOCS) (n=1)^(14)^, and the assessment of speech and/or language (n=1)^(21)^ (Figure 3).

Caption: RF 1 = Positive family history of stuttering (with or without recovery); RF 2 = High frequency of disfluencies typical of stuttering; RF 3 = Duration of stuttering; RF 5 = Child's age at the onset of stuttering; FR 6 – Male sex; RF 7 = Speech tension (effort to speak); RF 8 = Negative social reaction to the child's speech (extended family, school, and others); RF 9 = Worse performance on standardized articulatory/phonological assessment; RF 10 = Complaint of stuttering; RF 12 = Impairment in speech intelligibility; RF 14 = Presence of physical concomitants (body and/or facial movements); RF 16 = Below-average language development; RF 17 = Presence of comorbidities; RF 25 = Severity of stuttering. Source: Prepared by the authors, 2025

Observational and self-report-based methods were the most commonly used in the studies analyzed, and the Stuttering Severity Instrument-3 (SSI-3) was the most frequently used in studies that used complementary methods. A tendency was observed across most studies (n=11) to combine multiple assessment methods in their data collection procedures. The combination of assessment methods was used both for screening for risk factors and for confirming the stuttering diagnosis in the studied population.

In studies that used standardized methods for stuttering screening in preschoolers, only two formal instruments were identified, both Brazilian: the Protocolo de Risco para a Gagueira do Desenvolvimento (PRGD - Developmental Stuttering Risk Protocol)^(18,35)^; and the Instrumento de Rastreio para a Gagueira do Desenvolvimento (IRGD - Developmental Stuttering Screening Instrument)^(12,25)^. Studies that used the PRGD combined the instrument with a structured screening questionnaire for caregivers^(18)^, a speech fluency assessment^(18,35)^, and the application of the Stuttering Severity Instrument-3 (SSI-3)^(35)^. In comparison, the study that used the IRGD used only this instrument for stuttering screening in preschoolers as the sole data-collection procedure^(12)^. All assessment methods identified in this SR (except the standardized method using the IRGD) involve procedures that must be performed exclusively by the speech-language pathologist (Table 2).

It should also be noted that the search strategy used in this SR did not identify the recent screening instruments for stuttering in preschoolers, namely: the US instrument, Childhood Stuttering Screening for Physicians (CSS-P™)^(37)^; and the Chilean instrument, called Instrumento para la Detección Temprana de Tartamudez^(38)^. These standardized instruments were not included in the sample for two reasons: the CSS-P was not published in a journal that could be indexed in any database, only in a book chapter; and the article that published the Chilean instrument did not present descriptors compatible with those combined in the search strategies adopted in this SR, which made it impossible to retrieve them from the databases.

### Main limitations and suggestions mentioned in the reviewed literature

In general, the studies included in this SR shared limitations, mainly regarding sample size and representativeness, the absence of fully validated standardized instruments, and reliance on parental reports without direct clinical confirmation, factors that compromise the generalizability of the findings and may introduce bias. It is also highlighted the need to incorporate additional risk measures, since there is a possibility that risk estimators change with advancing age^(12-14,19-21,35)^, the necessity to carry out longitudinal follow-up of children to obtain the analysis of the permanent outcome, and the lack of a gold standard for diagnostic assessment for children in the risk group (Table 2).

The suggestions identified emphasize the need for methodological strengthening, through the expansion of samples and age diversification of children, the performance of more robust psychometric analyses, the application of instruments for stuttering screening by different professionals, and their exploration in digital formats, to facilitate multiprofessional use and collective screening of children at risk for stuttering^(12,21)^. The improvement of existing instruments was also suggested, either through the incorporation of new variables, the empirical validation of theoretical models, or the refinement of the severity and impact scales of stuttering (Table 2).

## DISCUSSION

Identifying risk factors for childhood-onset fluency disorders is relevant for clinical decision-making regarding diagnosis and the design of preventive and therapeutic approaches. Therefore, this SR analyzed and synthesized 11 scientific articles published in the international literature to map the state of the art on risk factors and assessment methods used in screening for fluency disorders in preschoolers.

### Absence of risk factors for cluttering in preschoolers?

Although the electronic search strategies adopted in this SR included the descriptor “cluttering”, no studies focused on screening and/or risk assessment for cluttering in preschool children were identified. Therefore, all the results of this SR refer to risk factors for stuttering and to the assessment methods used to screen for this fluency disorder in preschoolers. This result might be associated with the lack of agreement regarding the diagnostic criteria for cluttering, the scarcity of consolidated scientific evidence, and the later diagnosis of this disorder^(39,40)^.

However, it is observed that some risk factors identified in this SR are compatible with clinical characteristics observed in cluttering, among them: male sex^(11)^, alteration in speech rate, impairment in speech intelligibility, pneumophonoarticulatory incoordination^(12,18,25,35)^, presence of other disfluencies (repetition of non-monosyllabic words)^(12,25)^, and presence of comorbidities^(11,18,19,35)^. Since cluttering is also a fluency disorder that begins in childhood, the question arises: could the observation of these risk factors for stuttering also screen for cluttering risk in preschoolers? If so, some results from this screening may indicate a false-positive risk of stuttering.

Thus, children at risk for cluttering could be identified as children at risk for stuttering. The identification of common risk factors for stuttering and cluttering in assessment methods exclusively for stuttering screening underscores the need to update formal instruments for screening fluency disorders with onset in childhood. For example, a study that aimed to explore the applicability of the IRGD in the educational context concluded that the instrument's application enabled the identification of preschoolers at risk for fluency disorders, not just for stuttering^(41)^.

### The value of the stuttering complaint reported by parents/guardians

The high frequency of typical stuttering disfluencies in children's speech has been identified as an important risk factor for chronic stuttering by most of the studies analyzed^(12-14,19-21,25,35)^. Many researchers and clinicians describe stuttering as characterized by prolongations, blocks, and repetitions of speech^(6,42)^, and that a percentage of typical stuttering disfluencies ≥ 3% is a risk indicator for persistent stuttering^(43)^. However, the isolated analysis of this characteristic is not sufficient to establish the diagnosis, as certain populations, such as bilingual children, may yield false-positive diagnoses of stuttering^(44)^. In addition, stuttering shows high variability in its manifestations and may exhibit different disfluency frequencies depending on the interlocutors involved in different communicative moments and situations^(45)^.

The presence of typical stuttering disfluencies in a child's speech raises concern among parents/guardians, who seek clinical care to confirm the diagnosis. In general, parents' and guardians' reports are highly accurate^(46)^. In this sense, the complaint reported by parents/guardians, based on the observation of the high frequency of typical stuttering disfluencies, is an indispensable tool for research and clinical practice^(45)^. Among the most predictive risk factors for stuttering, the complaint was the most frequently cited in this SR^(12,13,18-20)^, which gives due weight to reports from parents/guardians and highlights the need to welcome and actively listen to families whose children have a complaint of stuttering.

### Stuttering, its genetic basis, and the importance of the time factor

Among the risk factors identified in the 11 studies analyzed, a positive family history of stuttering was the most frequent^(11-14,18,19,21,25,35,36)^ and considered one of the most predictive^(11,19,21)^. Stuttering is highly heritable, with distinct genetic variants shared among family members that impact the risk of the disorder^(6)^. Genetic transmission models of stuttering are consistent with high polygenicity, suggesting a complex genetic architecture^(6)^, but epigenetic processes influence gene expression throughout life^(47)^. Therefore, the growth patterns of the central nervous system and the final phenotype (e.g., stuttering versus non-stuttering) are not fixed at birth, but emerge throughout development, through the interaction of genes, epigenetic processes, and experience^(36,47)^.

Time, in turn, is a fundamental variable for stuttering, which has been considered in the field of Neuroscience as a neural timing disorder^(48)^, due to the presence of deficits in the temporal synchrony necessary for the precise coordination of electrical and chemical impulses in the central nervous system, fundamental for information processing. The onset of stuttering symptoms occurs during the neurodevelopmental period^(32)^. Therefore, it is necessary to identify the disorder as close to the onset of its symptoms as possible, so that appropriate speech therapy intervention is early and successful^(25,49,50)^. The more time passes for a child who stutters, the more their age and the duration of stuttering increase, while their chances of recovery decrease^(47)^, since the persistence of typical stuttering disfluencies for at least twelve months was a prevalent risk factor for most of the studies analyzed^(11-14,18,21,25,35)^.

### Does the reaction of others to the speech of a child who stutters matter?

Although both the child's negative reaction to their own speech^(11-13,18,25,35,36)^ and the negative social reaction to the child's speech (extended family, school and others)^(12,13,18,25,35,36)^ have been identified in the reviewed literature as risk factors for stuttering, the negative social reaction stands as the most predictive of stuttering^(12,13)^. This finding underscores the need to screen for stuttering risk within a broader care perspective, where the domains of contextual factors are considered alongside those of body functions and structures, according to the International Classification of Functioning, Disability and Health (ICF)^(51)^.

Identifying negative social reactions from family, school, and others regarding a child's speech as a predictive risk factor for stuttering underscores the environment's role during neurodevelopment, which is influenced by experience^(36,47)^. Furthermore, it emphasizes the importance of health education practices as tools for social transformation, capable of reducing the risk of triggering or maintaining stuttering among children. Therefore, care for children who stutter needs a broader perspective that also addresses contextual factors, including, for example, fluency-promotion guidelines for families, schools, and other social sectors.

### Impaired speech is a sign of a higher risk for stuttering: why?

Among the risk factors analyzed as most predictive of stuttering, the following were observed: poorer performance on standardized articulatory/phonological assessment^(14,21)^ and below-average language development^(14)^. But why would the presence of such linguistic alterations increase the risk of a preschooler stuttering? One possible explanation is to assume that stuttering is a fluency disorder that begins in childhood^(32)^ and that fluency is a linguistic skill^(52)^. According to the Teoria Integrada da Fluência (TIF - Integrated Fluency Theory), fluency is a skill of linguistic competence; that is, it is part of the set of mental capacities and skills that make any person capable of producing and understanding language^(52)^.

Both children and adults with stuttering present sensory-perceptual difficulties that reflect atypical patterns of development, whether in the production or comprehension of language^(53,54)^. The presence of structural alterations in the dorsal pathway of the language circuit in adults who stutter also provides support for the idea that stuttering is related to an impairment in the bidirectional mapping between the auditory and articulatory cortices, supported by the dorsal pathways^(55)^. The dorsal pathway is fundamental for the organization of words in sequence and for the generation of sentences^(56)^, as well as being specialized in the perceptual and phonological processing of spoken language through the analysis of acoustic complexity, disambiguation of sounds, and functional integration with auditory processing^(57)^.

Given this scenario, since language acquisition transitions from a prosodic and distributional analysis of speech material to a syntactic analysis of linguistic utterances, according to the phonological bootstrapping hypothesis^(58)^, the TIF hypothesizes that: since fluency is a linguistic skill, its typical development is directly related to optimal processes of identification and access to formal features^^1^^ , while stuttering, being a fluency disorder, is related to difficulties in the representation of formal features, mainly the categorical feature, in the mental lexicon^^2^^ and/or in accessing them during online computation^(52)^. Difficulties in these acquisitional requirements for language development can affect language comprehension and production, as well as hinder the initialization of the child's computational system^^3^^ , hindering the implementation of syntax and the process of categorical identification of words, as typical development requires^(60)^.

The conceptual proposal regarding what constitutes fluency and the hypotheses put forward by the TIF were experimentally verified, and the theory proved to be testable and potentially high in explanatory power, as the findings confirmed the hypotheses^(52)^. Therefore, the observation obtained in this SR, that risk factors related to impairments in language development are more predictive of stuttering^(14,21)^, is convergent with the theoretical assumption of fluency as a linguistic skill^(52)^. This fact should sensitize the speech-language pathology community to implement language assessment mechanisms in their practice with preschoolers who complain of stuttering, both in comprehension and in language production. Therefore, actions to screen for stuttering risk also involve investigating language alterations in child development.

### The scarcity of formal instruments for stuttering screening and Brazil's leading role in this area

This study identified a significant scientific gap, the scarcity of screening instruments for risk factors for fluency disorders in preschoolers. Among the standardized methods mapped, only two instruments were identified: the PRGD^(18,35)^ and the IRGD^(12,25)^. Both instruments were developed by Brazilian researchers, placing Brazil as a country scientifically committed to early screening for stuttering. This is possibly because the country's public health system has a national health promotion policy, aimed at building actions that enable it to respond to social health needs^(61)^. This policy encourages the development of health promotion and prevention actions at all levels of care, with emphasis mainly on primary care^(61)^.

In Brazil, health professionals working in primary health care and educators working in early childhood education are responsible for monitoring child development and identifying the first symptoms of changes in the child's health^(62,63)^. There is a bill in progress in the country^(64)^ that recognizes the need to guarantee children who stutter the right to early diagnosis and treatment. Therefore, the screening instruments to be applied in Brazil need to be multiprofessional, since child health care is decentralized. In this sense, the findings of this SR identified that only the IRGD demonstrated this characteristic.

The findings also showed that several assessment methods were combined to identify risk factors and diagnose stuttering in the studies analyzed. This information corroborates previous descriptions of stuttering as a multifactorial disorder, for which a multidimensional assessment is most appropriate to determine the factors that may be contributing to the instability observed in the child's fluency^(21,36,47)^.

### A single risk factor is not enough

There is a tendency to analyze risk factors in association, that is, the analysis of multiple domains is advocated in the assessment of a child's risk of developing persistent stuttering, since this point was made by most studies^(11,13,14,19-21)^. Analyzing a risk factor in isolation is not considered a predictive indicator of stuttering, as children may present different risk factor profiles^(20,21)^. Studies have shown that, in isolation, the complaint and the degree of severity, for example, were not predictive of eventual persistence or recovery in preschoolers^(20,21)^. When the analysis was carried out considering multiple factors, the presence of at least two predictive factors was significantly associated with a high risk of developing persistent stuttering^(14,21)^.

The identified risk factors can be causal, triggering, or maintaining factors of stuttering, and predictive risk analysis in preschool children should be cumulative, meaning that multiple risk factors should be considered^(14,19,20)^, and not just one factor in isolation. Experts recommend that the greater the number of identified risk factors, the earlier the child should be referred for specialized care^(11,14)^. Therefore, instruments for stuttering screening should be developed from a multifactorial perspective, with items covering multiple domains, so that the risk analysis is cumulative and personalized to each child's profile.

### Present and future of fluency disorder screening

The current landscape of fluency disorder screening is marked by two significant gaps: the lack of studies investigating risk factors for cluttering in preschoolers and the scarcity of standardized instruments for stuttering screening in this population, with assessment methods still predominantly dependent on speech-language pathologists. In contrast, a wide diversity of risk factors described in the literature is observed, which reveal significant advances by integrating, in addition to biological factors, clinically relevant contextual variables, such as personal factors (inherent to the child) and environmental factors (inherent to the extended family, school, and others).

The perspectives for future studies should prioritize the development and validation of screening instruments for fluency disorders, by including cluttering as a direct target disorder in early investigation, expanding and diversifying the sample, and incorporating digital technologies that enable large-scale early screening by different professionals. These advances are fundamental to improving the assessment methods used, thereby providing greater accuracy in screening for fluency disorders in early childhood. In addition to promoting both early identification and timely access to healthcare for children with fluency disorders.

### Limitations, practical recommendations, and clinical implications of this scoping review

This scoping review had limitations, including the limited number of databases consulted and the heterogeneity of the study samples, which made it difficult to compare results. There was also a concentration of publications from certain research groups, resulting in repeated methodologies and similar risk factors. The predominance of studies on stuttering, with little investigation of cluttering, limits the generalizability of the findings and underscores the need to expand the evidence base on fluency disorders in early childhood.

Despite the limitations, the review makes important contributions by systematizing data that can guide the development and validation of screening instruments for fluency disorders in childhood. The main risk factors associated with the persistence of stuttering, along with the critical analysis of assessment methods, stand out, offering practical support for the early identification and clinical monitoring of preschool children.

The findings of this review reinforce that early stuttering screening should consider several combined risk factors – such as parental complaints, family history, high frequency of typical stuttering disfluencies, duration, and the child's age at onset – which serve as warning signs for referral to speech-language pathology evaluation. The adoption of multidisciplinary screening protocols can broaden the scope of screening and promote earlier and more effective interventions.

We recommend that assessment instruments be updated and validated for different sociocultural contexts, incorporating variables related to cluttering and the ICF. The use of digital technologies to expand early detection and the development of research that considers the diversity of risk factors and populations are also beneficial, strengthening the existing empirical basis.

The results of this research have direct application in health and education practices, providing support for public policies and preventive actions to promote children's fluency. Screening based on multiple risk factors and family context supports prevention strategies and provides time for early, effective interventions.

## CONCLUSION

This scoping review identified evidence focused on risk factors and assessment methods for stuttering in preschoolers, but lacked specific studies on cluttering. The main risk factors included parental complaints, family history, high frequency of typical disfluencies, duration, and age of onset of stuttering. A scarcity of internationally standardized and validated instruments was found, with only two formal Brazilian protocols (PRGD and IRGD). The need to develop methods accessible to different professionals and contexts, as well as to expand early screening, is highlighted. We identified a scientific gap regarding cluttering and recommend studies that address its factors and assessment methods in childhood.

## Footnotes

1 Features are the values ​​and information encoded in the lexicon of a language, such that each lexical item consists of a composite of features. Formal features guide the computational system regarding the syntactic relationships that a given lexical item should establish with others within the sentence in which it is inserted. One of the functions of the categorical formal feature is to determine the distributional position of an item in the sentence^(59)^.

2 Mental lexicon, here, is conceived as a repository of knowledge of a phonological, semantic, pragmatic, and stylistic nature about linguistic items^(59)^.

3 A computational system, also known as mental grammar or syntax, is responsible for extracting information from the mental lexicon to derivatively construct syntactic representations through computational operations^(59)^.
