# Supplementary material for: Risk factors and assessment methods for screening childhood-onset fluency disorders: a scoping review
Source: Codas. 2026 Jul 6;38(4):e20250336. doi: 10.1590/2317-1782/e20250336en (PMC13343792; doi:10.1590/2317-1782/e20250336en)
Supplement: Translation

# Translation: Fatores de risco e métodos avaliativos para rastreamento dos transtornos da fluência com início na infância: uma revisão de escopo

**DOI:** 10.1590/2317-1782/e20250336pt

**Authors:** Mayra Maria Oliveira de Lima, Heloísa de Oliveira dos Anjos, Thaíse Sara Costa Dias, Débora Vasconcelos Correia, Cristiane Moço Canhetti de Oliveira

**Affiliations:** 1 Programa de Pós-graduação em Ciências da Saúde e Comunicação Humana, Universidade Estadual Paulista “Júlio de Mesquita Filho – UNESP - Marília (SP), Brasil.; 2 Programa de Pós-graduação em Linguística, Universidade Federal da Paraíba – UFPB - João Pessoa (PB), Brasil.

**Keywords:** Fatores de Risco, Gagueira, Transtorno da Fluência com Início na Infância, Desenvolvimento Infantil, Saúde da Criança, Fonoaudiologia, Revisão de Escopo

## Abstract

**Objetivo:**

Mapear o estado da arte sobre os fatores de risco e métodos avaliativos utilizados no rastreamento dos transtornos da fluência em pré-escolares.

**Estratégia de pesquisa:**

Revisão de escopo conforme o Instituto Joanna Briggs e checklist PRISMA-ScR. A questão norteadora foi: Quais os fatores de risco e métodos avaliativos utilizados no rastreamento dos transtornos da fluência (gagueira e taquifemia) em pré-escolares na literatura internacional? A estratégia PCC orientou a busca com descritores MESH e termos livres nas bases Lilacs, Pubmed/Medline, Scopus e ScienceDirect, entre 2020 e 2025, sem restrição de idioma.

**Critérios de seleção:**

Incluíram-se artigos originais disponíveis na íntegra que respondessem a pelo menos uma questão de pesquisa. Excluíram-se publicações não científicas, secundárias, sem resumo, fora do escopo conceitual e populacional, ou com co-ocorrência de outros transtornos.

**Análise dos dados:**

Os dados foram analisados descritivamente e sintetizados narrativamente.

**Resultados:**

Identificaram-se 2.091 publicações, após remoção de duplicatas e aplicação dos critérios, 11 artigos foram incluídos. Não foram encontrados estudos sobre fatores de risco para taquifemia. Para a gagueira, mapeou-se 14 fatores de risco mais preditivos relacionados às funções e estruturas do corpo e fatores contextuais. Apenas dois instrumentos padronizados de rastreamento foram identificados, ambos brasileiros: Protocolo de Risco para Gagueira do Desenvolvimento e Instrumento de Rastreio para a Gagueira do Desenvolvimento.

**Conclusão:**

Estudos sobre fatores de risco para taquifemia em pré-escolares mostram-se ausentes. Para a gagueira, fatores biológicos e contextuais se destacam. A maioria dos métodos avaliativos disponíveis são limitados ao uso do fonoaudiólogo, evidenciando lacunas para pesquisas futuras.

## INTRODUÇÃO

Os transtornos da fluência com início na infância mais conhecidos são a gagueira e a taquifemia. A gagueira é caracterizada principalmente por disfluências involuntárias, e esses sintomas primários comprometem o fluxo habitual e automático da fala^(1)^. Além disso, podem estar presentes comportamentos secundários, como a substituição ou evitação de palavras, na tentativa de minimizar as dificuldades na fluência^(2)^. A taquifemia é caracterizada pela velocidade de fala percebida como rápida e/ou irregular, acompanhada de, no mínimo, um dos seguintes sintomas: aumento na ocorrência das disfluências, sendo que a maioria não é típica da gagueira; coarticulação entre os sons; e pausas em posições atípicas^(3)^.

Ambos os transtornos acometem os padrões de temporalização da fala, possuem forte componente genético com característica de herdabilidade e predominância de persistência no sexo masculino^(4-6)^. Além disso, a gagueira e a taquifemia podem se apresentar de forma isolada ou coexistir^(7)^. Apesar da prevalência dos transtornos da fluência com início na infância afetar cerca de 5% da população pré-escolar^(8)^, nos casos de gagueira, e até 1,2% em adolescentes nos casos de taquifemia^(9)^, seus diagnósticos ainda são desafiadores e costumam ser tardios.

Por essa razão, especialistas orientam que a etapa inicial fundamental da avaliação da fluência infantil é investigar cuidadosamente os fatores de risco para o desenvolvimento desses transtornos^(10,11)^. Quanto mais fatores de risco a criança apresentar, maior a chance de a criança precisar de monitoramento especializado^(12-14)^. Diretrizes clínicas da Fonoaudiologia recomendam a avaliação dos fatores de risco para os transtornos da fluência com início na infância em crianças menores de seis anos, como base para tomadas de decisão^(15,16)^.

Fatores de risco são marcadores de predição de um problema de saúde futuro, importantes para orientação de decisões diagnósticas e terapêuticas^(17)^. Na gagueira, dentre os vários fatores de risco identificados na literatura, podemos citar: sexo masculino, queixa por parte dos responsáveis, presença de histórico familial, idade de início do transtorno e tempo de duração da queixa, entre outros^(11-14,18-21)^. Na taquifemia, alguns fatores de risco são semelhantes à gagueira, como sexo e o histórico familial, mas diferencia-se ao considerar o planejamento da linguagem desorganizado e a ausência ou baixa percepção da dificuldade comunicativa^(22,23)^.

No entanto, apesar de ambas as condições serem transtornos da fluência com início na infância, não encontram-se disponíveis instrumentos formais que auxiliem nos seus rastreamentos de maneira conjunta. Como a gagueira é o transtorno da fluência mais estudado, nota-se um esforço da comunidade científica em rastreá-la, a exemplo dos instrumentos: Protocolo de Risco para a Gagueira do Desenvolvimento (PRGD)^(24)^ e o Instrumento de Rastreio para a Gagueira do Desenvolvimento (IRGD)^(25)^. Todavia, o mesmo não se observa em relação à taquifemia, pois não há instrumentos para identificá-la precocemente na população pré-escolar.

Observa-se que os métodos avaliativos predominantemente utilizados no rastreamento dos fatores de risco para gagueira e taquifemia preconizam a avaliação clínica especializada, com instrumentos de uso exclusivo do fonoaudiólogo, a exemplo do PRGD^(24)^ - para pré-escolares e escolares, e do *Cluttering Inventory-Revised* (PCI-r)^(26)^, para escolares e adolescentes, o que inviabiliza ações multiprofissionais de rastreamento. Ao considerar que o sistema de saúde público brasileiro estabelece políticas de rastreamento para alterações comunicativas na primeira infância^(27-29)^, ainda não está claro quais fatores de risco pode-se considerar para o rastreamento da gagueira e da taquifemia em pré-escolares, bem como quais seriam os métodos avaliativos mais adequados para tal investigação.

Diante desse panorama, faz-se necessário identificar e sintetizar as pesquisas disponíveis sobre fatores de risco e métodos avaliativos para rastreamento dos transtornos da fluência com início na infância. Tal necessidade visa obter uma visão geral sobre o tema, identificar lacunas, tendências e termos comuns, o que se mostra compatível com uma abordagem de revisão da literatura do tipo escopo. Assim, lança-se a seguinte questão de pesquisa: Quais os fatores de risco e métodos avaliativos utilizados no rastreamento dos transtornos da fluência (gagueira e taquifemia) em pré-escolares na literatura internacional? O objetivo desta revisão de escopo foi mapear o estado da arte sobre os fatores de risco e métodos avaliativos utilizados no rastreamento dos transtornos da fluência em pré-escolares.

## MÉTODO

### Protocolo e registro

Esta revisão de escopo (RE) da literatura foi desenvolvida conforme a metodologia proposta pelo Instituto Joanna Briggs (JBI), em consonância com as recomendações do guia *Reporting Items for Preferred Systematic Reviews and Meta-Analyses extension for Scoping Reviews* (PRISMA-ScR). Uma das indicações para se realizar a RE é quando se pretende identificar as principais características ou fatores relacionados a um conceito^(30)^, assim como na presente pesquisa. O protocolo desta RE foi registrado na plataforma Open Science Framework^(31)^.

### Questões de pesquisa

Para orientar a formulação da questão norteadora desta RE, adotou-se a estratégia População, Conceito e Contexto (PCC). Assim, definiu-se como: população, criança pré-escolar com transtorno da fluência (gagueira e/ou taquifemia), com idade entre 2 a 5:11 anos; conceito: fatores de risco e avaliação/rastreamento; e contexto: literatura científica internacional. Logo, obteve-se a seguinte questão de pesquisa (QP) primária: Quais os fatores de risco e métodos avaliativos utilizados no rastreamento dos transtornos da fluência em crianças pré-escolares na literatura internacional? Como questões de pesquisa secundárias elegeu-se: Quais os fatores de risco identificados? (QP1); Quais os métodos avaliativos utilizados? (QP2); Quais as limitações descritas nos estudos? (QP3); Quais as sugestões mencionadas pelos autores para novas pesquisas na área? (QP4).

### Critérios de elegibilidade

Foram excluídas as publicações que não se classificaram como artigos científicos, tais como: cartas ao editor, capítulos de livros, resumos de anais de eventos científicos, artigos de opinião; artigos secundários (revisões da literatura); artigos sem resumo; artigos fora do escopo conceitual e populacional de interesse; ou com co-ocorrência de outros transtornos, tais como: deficiência intelectual, disfunção neuromotora, transtorno do déficit de atenção e hiperatividade (TDAH), transtorno do espectro do autismo (TEA), e trissomia do cromossomo 21 (Síndrome de Down). Não houve exclusão com base no idioma, etnia, raça, sexo, gênero e localização geográfica.

Foram incluídos, portanto, os artigos científicos disponíveis na íntegra; publicados entre 2020 e 2025; e os artigos que responderam a pelo menos uma questão de pesquisa. Incluiu-se também artigos técnicos e empíricos que propuseram discutir modelos teóricos e/ou diretrizes clínicas de avaliação dos fatores de risco para a população de interesse.

Delimitou-se a idade das crianças até 5:11 anos por corresponder ao período crítico de surgimento dos transtornos da fluência com início na infância^(32)^. O recorte temporal das publicações foi delimitado nos últimos cinco anos para que as informações coletadas fossem sobre as evidências científicas mais recentes da literatura. Apenas os artigos disponíveis na íntegra foram selecionados, possibilitando a coleta de dados para a RE e o alcance do objetivo proposto. Optou-se por incluir os artigos técnicos e empíricos para ampliar os dados sobre fatores de risco e entender as diretrizes recomendadas para a avaliação desses fatores. Excluiu-se a presença de outros transtornos em co-ocorrência com os transtornos da fluência para que os fatores de risco e métodos avaliativos identificados fossem relacionados exclusivamente à população de interesse, ou seja, crianças pré-escolares com diagnóstico ou risco para a gagueira e a taquifemia.

### Bases de dados

As bases de dados foram: Lilacs (Literatura Latino-Americana e do Caribe em Ciências da Saúde), *Medical Literature Analysis and Retrieval System* (Pubmed/Medline), Scopus e ScienceDirect. A busca foi realizada em 30 de junho de 2025. A plataforma *ResearchGate* foi utilizada para realizar busca ativa junto aos autores dos artigos selecionados não disponíveis na íntegra após a etapa de triagem, a fim de preservar a amostragem dos estudos triados e selecionados para a leitura e análise do texto completo na etapa de elegibilidade. Essa busca ativa ocorreu em 31 de julho de 2025.

### Estratégias de busca

Inicialmente identificou-se as palavras mais utilizadas e frequentemente citadas nos manuais diagnósticos e diretrizes clínicas da área, para referir-se aos conceitos e populações de interesse desta RE. Definiu-se, portanto, como estratégia de busca eletrônica, as combinações entre descritores MESH (*Medical Subject Headings*) e termos livres, em inglês, considerados relevantes para a pesquisa, adaptando-se aos padrões de busca estabelecidos em cada base de dados, conforme disposto no Quadro 1.

**Quadro 1:** Apresentação das estratégias de busca nas bases de dados

**Base de dados**	**Estratégia de busca**
Lilacs, Pubmed/Medline e ScienceDirect (via Portal CAPES)	((child OR "early childhood" OR preschool) AND (stuttering OR stammering OR cluttering OR "fluency disorder") AND ("risk factor" OR assessment OR screening))
Scopus (via Portal CAPES)	((child OR "early childhood" OR preschool OR children) AND (stuttering OR stammering OR cluttering OR "fluency disorder") AND ("risk factor*" OR "predisposing factor*" OR "contributing factor*") AND (assessment OR screen*))

Fonte: Dos autores, 2025

A validação da estratégia de busca seguiu as etapas recomendadas pelo Instituto Joanna Briggs e checklist PRISMA-ScR: 1) busca preliminar dos artigos, a fim de verificar a sensibilidade e especificidade da estratégia; 2) leitura de alguns resumos de artigos com potencial para inclusão na revisão; e 3) validação por uma fonoaudióloga especialista em Fluência, não envolvida na equipe de pesquisa. Dessa forma, foi possível identificar necessidades de ajustes na combinação dos termos de busca, obter sugestões de melhorias e concluir a validação da estratégia de busca escolhida.

### Seleção dos estudos

A seleção dos artigos ocorreu em quatro etapas (Figura 1): identificação; triagem; elegibilidade e inclusão. Para a realização das três primeiras etapas utilizou-se o software Rayyan^(33)^. A pesquisadora principal coordenou o uso do software e foi responsável pelo treinamento de duas revisoras independentes, ambas fonoaudiólogas doutorandas com expertise na área da Fluência. As revisoras participaram das etapas de triagem, elegibilidade e inclusão. O treinamento das revisoras foi realizado na etapa de triagem, com quarenta e dois artigos. Após o treinamento iniciou-se a RE. As decisões sobre eventuais discordâncias foram resolvidas por consenso, a fim de garantir a qualidade dos processos decisórios, e contou com a participação de uma terceira revisora quando se fez necessário.

**Figura 1:** Fluxograma da estratégia de seleção dos artigos

Fonte: Dos autores, 2025

A primeira etapa (identificação) foi realizada pela pesquisadora principal, mediante o controle das publicações duplicadas e identificação quantitativa dos artigos a serem analisados por bases de dados. A segunda etapa (triagem) deu-se por meio da aplicação dos critérios de exclusão, mediante a leitura do título, resumo e palavras-chave. E a terceira etapa (elegibilidade) ocorreu por meio da análise dos critérios de inclusão, mediante a leitura do artigo completo.

### Extração, análise e síntese dos dados

Na quarta etapa, da inclusão, realizou-se a extração das respostas às questões de pesquisa de cada artigo selecionado. Um formulário em planilha digital do Microsoft Excel, elaborado e calibrado previamente pela equipe, foi utilizado para a extração de dados. O preenchimento ocorreu de forma independente e cega pelas revisoras treinadas e, posteriormente, consolidado pela pesquisadora principal. As variáveis para as quais coletou-se dados em cada etapa da RE foram:

1. *Identificação:* total de publicações identificadas; total de publicações identificadas por base de dados; total de publicações duplicadas; total de publicações selecionadas por base de dados (após a eliminação das duplicações).
2. *Triagem:* tipo de publicação; possibilidade de tratar sobre os conceitos e população de interesse; presença de comorbidade(s) na população de interesse.
3. *Elegibilidade e Inclusão:* disponibilidade do artigo na íntegra; fatores de risco; métodos avaliativos; limitações descritas; sugestões apontadas.
4. *Caracterização dos artigos incluídos:* título; ano de publicação; base de dados; periódico; autor(es); objetivo; idioma; país; público-alvo; idade; tamanho da amostra (se aplicável); tipo da pesquisa (conforme o nível de evidência científica).

Cabe pontuar que, especificamente sobre as variáveis fatores de risco e métodos avaliativos, extraídas nas etapas de elegibilidade e inclusão, fez-se necessário o processo de padronização terminológica para a análise e síntese dos resultados. Os termos utilizados para designar os fatores de risco e os métodos avaliativos utilizados nos artigos incluídos foram agrupados pela equipe a partir dos seguintes critérios: 1) similaridade de sentido, e; 2) seleção de termos preferenciais, preconizando os mais frequentes e atuais na literatura. Assim, foi possível elaborar um vocabulário controlado da revisão em tabela e utilizar os termos padronizados na síntese dos resultados.

Apesar de não ter sido realizada a avaliação crítica dos artigos incluídos, em consonância com os objetivos de revisões de escopo, todos os artigos foram caracterizados quanto ao seu nível de evidência por meio da *Agency for Healthcare and Research and Quality* (AHRQ)^(34)^, a fim de explicitar a sua qualidade metodológica. Os resultados foram analisados descritivamente, sintetizados narrativamente e dispostos em figuras e tabelas. As implicações para a prática clínica e pesquisas futuras também foram discutidas.

## RESULTADOS

### Seleção dos estudos

Por meio da busca eletrônica nas bases de dados foi possível recuperar 2.091 publicações, sendo 15 na Lilacs, 1.508 na PubMed/Medline, 206 na Scopus e 362 na ScienceDirect. Foram excluídas 91 publicações duplicadas. Foram triadas 2.000 publicações por títulos, resumos e palavras-chave. Após a aplicação dos critérios de exclusão, 1.934 publicações foram excluídas. Selecionou-se 66 artigos para a leitura completa, destes, após a aplicação dos critérios de inclusão, 55 artigos foram excluídos. Ao término da etapa de elegibilidade 11 artigos foram incluídos na RE para análise, síntese qualitativa e descrição dos resultados (Figura 1).

### Caracterização dos artigos incluídos

Obteve-se cinco artigos da base de dados Scopus, quatro da Lilacs, um da PubMed/Medline e um da ScienceDirect. Observou-se que seis estudos foram brasileiros^(12,13,18,20,25,35)^, dois estadunidenses^(14,21)^, dois holandeses^(11,36)^ e um japonês^(19)^, cujos anos de publicação variaram entre 2021 e 2025. Apenas três artigos se configuraram como estudos teóricos (níveis VI e VII de evidência)^(11,25,36)^, os demais^(12-14,18-21,35)^ distribuíram-se do nível III ao nível VI de classificação dos níveis de evidência científica proposto pela *Agency for Healthcare Research and Quality* (AHRQ)^(34)^. A idade da população estudada variou entre dois e doze anos, de ambos os sexos, com amostras que variaram entre 30 e 2.055 participantes. Todos os artigos incluídos consideraram crianças que gaguejam como população de interesse. Não se obteve nenhum estudo voltado para o rastreamento e/ou avaliação de risco para a taquifemia em pré-escolares (Tabela 1)

**Tabela 1:** Caracterização dos artigos incluídos na revisão de escopo

Autores (Ano)	Base de dados	Periódico	Objetivo	Nível de evidência	Transtorno da fluência rastreado	Amostra	Faixa etária (anos)	Idioma	Uso de algum instrumento para rastreamento?
						(se aplicável)		(País)	
Lima et al. (2021a)	Lilacs	Cefac	Elaborar um instrumento de rastreio com fins de identificação do risco para a gagueira do desenvolvimento em crianças pré-escolares	Observacional, analítica e transversal	Gagueira	NSA	2-5	Português (Brasil)	Sim
				Nível VI					
Lima et al (2021b)	Lilacs	Cefac			Gagueira	30	2-5	Português (Brasil)	Sim
			Verificar a sensibilidade e as medidas de acurácia do IRGD	Observacional, analítica e transversal					
				Nível VI					
Pinto et al. (2021)	Lilacs	Audiology Communications Research	Testar a variável da hereditariedade familiar para a GCD como preditora de efeito direto no desfecho da fluência da fala em crianças		Gagueira	200	2-12	Português (Brasil)	Sim
				Caso-controle					
				Nível IV					
Walsh et al. (2021)	Scopus	Journal of Speech, Language and Hearing Research	Investigar como fatores epidemiológicos e clínicos preveem coletivamente se uma criança em idade pré-escolar que gagueja persistirá ou se recuperará	Coorte	Gagueira	52	3-5	Inglês	Sim
			e fornecer orientação sobre como os médicos podem usar esses fatores para avaliar o risco de uma criança gaguejar e persistir	Nível IV				(EUA)	
Ávila et al (2022)	Lilacs	CoDAS	Delinear um ensaio clínico de tratamento – em três modalidades – que verificasse se os tratamentos testados apresentam indicadores que permitam reunir informações para a continuidade da sua aplicação, estabelecendo uma relação benefício-risco eficaz e segura	Ensaio clínico não randomizado	Gagueira	93	2-12	Português (Brasil)	Sim
				Nível III					
Costa et al. (2022)	Scopus	Int. J. Environ. Res. Public Health	Correlacionar as características da fluência da fala de crianças cujos pais relataram comportamentos de gagueira com os fatores de risco da gagueira persistente	Observacional e prospectivo	Gagueira	417	2-11	Português (Brasil)	Não
				Nível VI					
Singer et a (2022)	Scopus	Journal of Speech, Language, and Hearing Research	Avaliar se a presença de um número crescente de fatores preditivos aumenta o risco de uma criança desenvolver gagueira persistente	Documental e retrospectivo	Gagueira	67	3-5	Inglês	Sim
				Nível VI				(EUA)	
	
			Abordar todas as etapas que um fonoaudiólogo deve seguir, de acordo com as Diretrizes de Gagueira revisadas (NVLF, 2020), com uma criança pequena que gagueja, com foco na avaliação dos fatores de risco e na implementação do monitoramento.						
								
Tonnis, Ormondt (2022)	Scopus	Stem-Spraak-		Narrativo e descritivo	Gagueira	NSA	< 6	Holandês	Não
		En taalpathologie		Nível VII				(Holanda)	
								
Costa et al. (2023)	PubMed/Medline	CoDAS	Investigar duas variáveis independentes consideradas como dois possíveis preditores de risco cumulativo para gagueira persistente: percepção familiar da gagueira e quantidade de rupturas na fala.	Estudo observacional, transversal e retrospectivo	Gagueira	452	3-11	Português (Brasil)	Sim
				Nível VI					
Franken et al. (2024)	Science Direct	Journal of Fluency Disorders	Atualizar e expandir o modelo clínico Erasmus, incorporando evidências recentes para explicar o início, o desenvolvimento e os possíveis desfechos da gagueira em criança.	Estudo teórico de caráter narrativo com proposição de modelo clínico	Gagueira	NSA	Pré-	Inglês	Não
				Nível VI			escolar	(Holanda)	
Sakai et al (2025)	Scopus	Folia Phoniatrica et Logopaedica	Investigar a prevalência, a incidência cumulativa e os fatores de risco da gagueira na primeira infância no Japão	Observacional, transversal e epidemiológico	Gagueira	2.055	3		
				Nível VI				Inglês	
								(Japão)	Sim

**Legenda:** EUA = Estados Unidos da América; NSA = Não se aplica. Fonte: Dos autores, 2025

### Fatores de risco para a gagueira em pré-escolares

Após a padronização dos termos utilizados nos artigos para designar os fatores de risco, foram identificados 27 fatores considerados de risco para a gagueira na população pré-escolar. Os dez fatores de risco mais citados foram: histórico familial positivo para a gagueira (com ou sem recuperação) (n=10)^(11-14,18,19,21,25,35,36)^; alta frequência de disfluências típicas da gagueira (n=8)^(12-14,19-21,25,35)^; tempo de duração da gagueira (n=8)^(11-14,18,21,25,35)^; reação negativa da criança em relação à própria fala (n=7)^(11-13,18,25,35,36)^; idade da criança no início da gagueira (n=6)^(11,13,14,19,21,35)^; sexo masculino (n=6)^(11,13,14,19,21,35)^; tensão na fala (esforço para falar) (n=6)^(12,13,18,19,25,35)^; reação social negativa em relação à fala da criança (família estendida, escola e outros) (n=6)^(12,13,18,25,35,36)^; pior desempenho em avaliação articulatória/fonológica padronizada (n=6)^(11,14,18,21,35,36)^; e queixa de gagueira (n=5)^(12,18-20,25)^ (Tabela 2).

**Tabela 2:** Matriz dos resultados da revisão de escopo

Autores (Ano)	QP1		QP2	QP3		QP4
	Fatores de risco identificados	Fatores de risco mais preditivos (desfechos)	Métodos avaliativos	Limitações	Sugestões
	16			Limitações ao próprio processo de construção de instrumentos por se encontrar em fase inicial do processo geral de validação. As evidências de validade de conteúdo são essenciais, mas não são suficientes.	A continuidade das análises psicométricas por meio do emprego de novas medidas de acurácia.
Lima et al. (2021a)	(FR 1, FR 2, FR 3, FR 4, FR 7, FR 8, FR 10, FR 11, FR 12, FR 13, FR 14, FR 15, FR 19, FR 20, FR 21, FR 22)	NSA	IRGD		
			
Lima et al. (2021b)	16	4		O tamanho amostral reduzido devido ao curto tempo de coleta dos dados atribuída à crise sanitária mundial, e a ausência de uma avaliação considerada “padrão ouro” da área para diagnosticar as crianças do grupo de risco.	(1) ampliação do tamanho da amostra; (2) comparação das crianças de risco com o diagnóstico clínico; (3) aplicação do instrumento por mais profissionais a fim de obter outras análises de validação psicométrica; (4) comparação da aplicação em formato digital
	(FR 1, FR 2, FR 3, FR 4, FR 7, FR 8, FR 10, FR 11, FR 12, FR 13, FR 14, FR 15, FR 19, FR 20, FR 21, FR 22)	(FR 8, FR 10, FR 12, FR 14)	IRGD		
	17	3			
Pinto et al. (2021)	(FR 1, FR 3, FR 4, FR 7, FR 8, FR 9, FR 10, FR 11, FR 12, FR 13, FR 14, FR 15, FR 16, FR 17, FR 18, FR 23, FR 24)	(FR 2, FR 5, FR 10)	PRGD	Não menciona	Não menciona
					
Walsh et al. (2021)					
				
	6	3	Métodos observacionais;	(1) incluir uma amostra maior de crianças com gagueira em diferentes idades e incorporar medidas de risco adicionais.	(1) criar uma calculadora online na qual os médicos possam inserir a idade da criança, fatores epidemiológicos relevantes e outras pontuações coletadas durante uma avaliação para determinar o risco coletivo de persistência da gagueira em cada criança
	(FR 1, FR 2, FR 3, FR 5, FR 6, FR 9)	(FR 1, FR 2, FR 9)	Métodos complementares: Avaliação da fala/linguagem.		
				
Ávila et al. (2022)				(2) Efeito do tamanho da amostra (grupos não homogêneos quanto ao número de participantes); ausência de randomização; controle apenas da variável % de sílabas gaguejadas, sem avaliar outras mudanças relacionadas à gagueira (variabilidade sazonal, fala fora da clínica, habilidades interativas, perfil emocional e ambiental, entre outros); (4) Não contemplou o seguimento a longo prazo das crianças impedindo a análise de desfecho permanente do quadro clínico.	
			
	19	NSA	PRGD		Não menciona
	(FR 1, FR 2, FR 3, FR 4, FR 5, FR 6, FR 7, FR 8, FR 9, FR 11, FR 12, FR 13, FR 14, FR 15, FR 16, FR 17, FR 18, FR 23, FR 24)		Métodos observacionais;		
			Métodos complementares: SSI-3		
			
	7	4	Métodos baseados em autorrelato;	(1) As crianças sem comportamentos de gagueira (NCSC) não apresentaram histórico familiar de gagueira. Talvez por isso o estudo não identificou a variável "histórico familiar" como preditor para gagueira;	Sugerem abranger diferentes faixas etárias para poder analisar o fator de risco de início tardio dos comportamentos de gagueira.
Costa et al. (2022)	(FR 1, FR 3, FR 4, FR 5, FR 6, FR 7, FR 8)	(FR 3, FR 7, FR 8, FR 10)	Métodos observacionais;	(2) a maioria das crianças neste estudo era mais jovem (a média de idade foi de 6,5 anos em ambos os grupos). Talvez por isso não foi possível verificar se os fatores de risco "queixa de gagueira por mais de 12 meses" e "queixa de gagueira antes dos 5 anos de idade" seriam preditores para a gagueira;	
				(3) os resultados deste estudo foram derivados de uma única instituição;	
				(4) A análise da fala se baseou exclusivamente nas primeiras 200 sílabas fluentes e não utilizou outras amostras de fala, como leitura oral, nomeação de palavras isoladas e amostras de fala mais longas.	
Singer et al. (2022)					(1) Incluir dados sobre o temperamento e a reação da criança à gagueira para explorar as consequências negativas relacionadas à gagueira (além da persistência) de forma mais abrangente;
			Métodos baseados em autorrelato;	(1) Disponibilidade de dados; não incluiu o domínio temperamento/emoção;	(2) incluir DTGs ponderadas;
	10	5	Métodos observacionais;	(2) As medidas empregadas não foram idênticas em todos os casos.	(3) Incluir o desempenho em uma tarefa de repetição de pseudopalavras;
	(FR 1, FR 2, FR 3, FR 5, FR 6, FR 9, FR 16, FR 18, FR 25)	(FR 3, FR 5, FR 9, FR 16, FR 25)	Métodos complementares: TOCS, SSI-3.		(4) Incluir o autorrelato da criança de estar ou não gaguejando ainda;
				
Tonnis e Ormondt (2022)	9	4	NSA	Não menciona	Não menciona
	(FR 1, FR 3, FR 4, FR 5, FR 6, FR 9, FR 16, FR 17, FR 26)	(FR 1, FR 3, FR 5, FR 6).			
				(1) Análise dos antecedentes familiares dos participantes;	
				(2) Diferenças nos percentuais e perfis das rupturas em grupos de meninos e meninas;	
Costa et al. (2023)	2	2	Métodos baseados em autorrelato;	(3) Tempo de início dos sintomas e considerações sobre as atitudes familiares.	Menciona que as limitações serão abordadas em estudos futuros.
	(FR 2, FR 10)	(FR 2, FR 10)	Métodos observacionais.		
					
					
Franken et al.	5	NSA	NSA	(1) O modelo é baseado em síntese teórica e revisão narrativa da literatura, portanto não tem validação empírica direta.	Validação empírica do modelo e aprimoramento das escalas de gravidade e impacto da gagueira.
-2024	(FR 1, FR 4, FR 8, FR 9, FR 27)			(2) Ele depende da integração de múltiplos fatores de risco (genéticos, neurológicos, linguísticos, emocionais, sociais), mas a relação causal entre esses fatores ainda não está totalmente comprovada;	
				(3) O modelo é mais útil como ferramenta clínica e de comunicação com famílias e pacientes do que como evidência científica de alta hierarquia.	
			Métodos baseados em autorrelato;	(1) Superestimativa da incidência e prevalência	Não menciona
Sakai et al. (2025)	7	3	Métodos observacionais;	(2) Redução da incidência e a prevalência estimadas pela evitação de alguns pais em confirmar a gagueira dos filhos pelo forte estigma social quanto ao questionário	
	(FR 1, FR 2, FR 5, FR 6, FR 7, FR 10, FR 17)	(FR 1, FR 10, FR 17)		(3) Base em relatos parentais, sem confirmação clínica direta	
				(4) Possibilidade de viés de memória nas respostas dos pais	
				(5) Como crianças com mais de quatro anos não estão incluídas na amostra desta pesquisa, não é possível capturar todos os casos de gagueira de início precoce na infância.	

**Legenda:** QP1 = Questão de pesquisa secundária 1; QP2 = Questão de pesquisa secundária 2; QP3 = Questão de pesquisa secundária 3; QP4 = Questão de pesquisa secundária 4; FR 1 = Histórico familial positivo para gagueira (com ou sem recuperação); FR 2 = Alta frequência de disfluências típicas da gagueira; FR 3 = Tempo de duração da gagueira; FR 4 = Reação negativa da criança em relação à própria fala; FR 5 = Idade da criança no início da gagueira; FR 6 = Sexo masculino; FR 7 = Tensão na fala (esforço para falar); FR 8 = Reação social negativa em relação à fala da criança (família estendida, escola e outros); FR 9 = Pior desempenho em avaliação articulatória/fonológica padronizada; FR 10 = Queixa de gagueira; FR 11 = Intercorrências pré, peri e pós-natal; FR 12 = Prejuízo na inteligibilidade do discurso; FR 13 = Incoordenação pneunofonoarticulatória; FR 14 = Presença de concomitantes físicos (movimentos corporais e/ou faciais); FR 15 = Alteração na velocidade de fala; FR 16 = Desenvolvimento da linguagem expressiva abaixo da média; FR 17 = Presença de comorbidades; FR 18 = Prejuízo nas habilidades de compreensão da linguagem; FR 19 = Presença de alergias (dermatite atópica, alergia alimentar, asma, e rinosinusite); FR 20 = Presença de alterações no sono (insônia, ronco, bruxismo, agitação noturna); FR 21 = Presença de outras disfluências (repetição de palavra não monossilábica); FR 22 = Demonstração de percepção da gagueira (a criança percebe que gagueja); FR 23 = Tipo de surgimento persistente da gagueira; FR 24 = Componentes estressantes da qualidade de vida; FR 25 = Grau de severidade da gagueira; FR 26 = Desenvolvimento da linguagem acima da média; FR 27 = Alterações neuroanatomofuncionais; IRGD = Instrumento Risco para a Gagueira do Desenvolvimento; PRGD = Protocolo de Risco para a Gagueira do Desenvolvimento;.TOCS = Test of Childhood Stuttering; SSI-3 = Stuttering Severit Instrument; NSA = Não se aplica. Fonte: Dos autores, 2025

Dos onze artigos incluídos, oito se dedicaram a investigar quais fatores de risco se mostraram mais preditivos para a gagueira. Assim, dos 27 fatores de risco identificados, 14 se destacaram como sendo os mais preditivos para a gagueira. A seguir, os sete fatores de risco mais citados como sendo os mais preditivos para a gagueira foram: queixa de gagueira (n=5)^(12,13,18-20)^; histórico familial positivo para a gagueira (com ou sem recuperação) (n=3)^(11,19,21)^; idade da criança no início da gagueira (n=3)^(11,14,18)^; tempo de duração da gagueira (n=3)^(11,13,14)^; alta frequência de disfluências típicas da gagueira (n=3)^(18,20,21)^; reação social negativa em relação à fala da criança (família estendida, escola e outros) (n=2)^(12,13)^; e pior desempenho em avaliação articulatória/fonológica padronizada (n=2)^(14,21)^ (Figura 2).

**Figura 2:** Consolidado dos métodos avaliativos no rastreamento de crianças pré-escolares que gaguejam

Fonte: Dos autores, 2025

### Métodos avaliativos utilizados para rastrear a gagueira em pré-escolares

Após a padronização de termos utilizados nos artigos para designar os métodos avaliativos utilizados no rastreamento da gagueira em pré-escolares, observou-se quatro grupos de métodos, sendo eles: métodos observacionais (n=7)^(13,14,18-21,35)^, baseados nos procedimentos necessários para a avaliação da fluência na fala; métodos baseados em autorrelato (n=6)^(13,14,18-21)^, mediante a aplicação de questionário estruturado de triagem aos responsáveis; métodos padronizados para o rastreamento da gagueira (n=4)^(12,18,25,35)^, baseados na utilização de instrumentos formais para esse objetivo; e métodos complementares (n=3)^(14,21,35)^, baseados no uso de escalas de gravidade perceptiva-clínica da gagueira (n=1)^(21)^, na aplicação do *Stuttering Severity Instrument-*3 (SSI-3) (n=2)^(14,35)^, do *Test of Childhood Stuttering* (TOCS) (n=1)^(14)^, e da avaliação da fala e/ou linguagem (n=1)^(21)^ (Figura 3).

**Figura 3:** Frequência dos Fatores de Risco mais preditivos para a Gagueira Persistente

Legenda: FR 1 = Histórico familial positivo para gagueira (com ou sem recuperação); FR 2 = Alta frequência de disfluências típicas da gagueira; FR 3 = Tempo de duração da gagueira; FR 5 = Idade da criança no início da gagueira; FR 6 = Sexo masculino; FR 7 = Tensão na fala (esforço para falar); FR 8 = Reação social negativa em relação à fala da criança (família estendida, escola e outros); FR 9 = Pior desempenho em avaliação articulatória/fonológica padronizada; FR 10 = Queixa de gagueira; FR 12 = Prejuízo na inteligibilidade do discurso; FR 14 = Presença de concomitantes físicos (movimentos corporais e/ou faciais); FR 16 = Desenvolvimento da linguagem abaixo da média; FR 17 = Presença de comorbidades; FR 25 = Grau de severidade da gagueira. Fonte: Dos autores, 2025

Os métodos observacionais e os métodos baseados em autorrelatos foram os mais utilizados nos estudos analisados, assim como a utilização do *Stuttering Severity Instrument*-3 (SSI-3) foi a mais frequente nos estudos que fizeram uso de métodos complementares. Observou-se uma tendência, para a maioria dos estudos (n=11), em combinar mais de um método avaliativo nos procedimentos de coleta de dados. A combinação de métodos avaliativos foi utilizada tanto para o próprio rastreamento dos fatores de risco, quanto para a confirmação do diagnóstico de gagueira na população estudada.

Nos estudos que utilizaram métodos padronizados para o rastreamento da gagueira em pré-escolares, identificou-se apenas dois instrumentos formais, ambos brasileiros: o Protocolo de Risco para Gagueira do Desenvolvimento (PRGD)^(18,35)^; e o Instrumento de Rastreio para a Gagueira do Desenvolvimento (IRGD)^(12,25)^. Os estudos que utilizaram o PRGD fizeram uso combinado do instrumento com um questionário estruturado de triagem aos responsáveis^(18)^, avaliação da fluência na fala^(18,35)^ e aplicação do *Stuttering Severity Instrument*-3 (SSI-3)^(35)^. Ao passo que o estudo que fez uso do IRGD utilizou apenas este instrumento para rastreamento da gagueira em pré-escolares como único procedimento de coleta de dados^(12)^. Cabe pontuar que todos os métodos avaliativos identificados nesta RE, exceto o método padronizado que utilizou o IRGD, envolvem procedimentos avaliativos que devem ser realizados exclusivamente pelo fonoaudiólogo (Tabela 2).

Cabe pontuar também que a estratégia de busca utilizada nesta RE não identificou os recentes instrumentos de rastreamento para a gagueira em pré-escolares, sendo eles: o instrumento estadunidense, *Childhood Stuttering Screening for Physicians* (CSS-P™)^(37)^; e o instrumento chileno, denominado *Instrumento para la Detección Temprana de Tartamudez* ^(38)^. Tais instrumentos padronizados não compuseram a amostra por duas razões: o CSS-P não foi publicado em periódico que pudesse estar indexado em alguma base de dados, apenas em capítulo de livro; e o artigo que publicou o instrumento chileno não apresentou descritores compatíveis com os combinados nas estratégias de busca adotadas nesta RE, o que impossibilitou a sua recuperação nas bases de dados.

### Principais limitações e sugestões mencionadas na literatura revisada

De modo geral, os estudos incluídos nesta RE apresentaram limitações convergentes, principalmente no tocante ao tamanho e à representatividade das amostras, à ausência de instrumentos padronizados plenamente validados e à dependência de relatos parentais sem confirmação clínica direta, fatores que comprometem a generalização dos achados e podem introduzir vieses. Também destacou-se a necessidade de incorporar medidas de risco adicionais, uma vez que há possibilidade de os estimadores de risco mudar com o avanço da idade^(12-14,19-21,35)^; de realizar acompanhamento longitudinal das crianças para obter a análise do desfecho permanente; e a falta de um padrão ouro de avaliação diagnóstica para as crianças do grupo de risco (Tabela 2).

As sugestões identificadas enfatizam a necessidade de fortalecimento metodológico, por meio da ampliação das amostras e diversificação etária das crianças, da realização de análises psicométricas mais robustas, da aplicação dos instrumentos para rastreamento da gagueira por diferentes profissionais e sua exploração em formatos digitais, a fim de facilitar o uso multiprofissional e o rastreamento coletivo das crianças com risco para a gagueira^(12,21)^. O aprimoramento de instrumentos existentes também foi sugerido, seja mediante a incorporação de novas variáveis, a validação empírica de modelos teóricos ou pelo refinamento das escalas de gravidade e impacto da gagueira (Tabela 2).

## DISCUSSÃO

A identificação dos fatores de risco para os transtornos da fluência com início na infância é relevante para a tomada de decisão clínica referente ao diagnóstico e delineamento de condutas preventivas e terapêuticas. Por isso, esta RE analisou e sintetizou onze artigos científicos, publicados em literatura internacional, com a finalidade de mapear o estado da arte sobre os fatores de risco e métodos avaliativos utilizados no rastreamento dos transtornos da fluência em pré-escolares.

### Ausência de fatores de risco para a taquifemia em pré-escolares?

Apesar das estratégias de busca eletrônica adotadas nesta RE incluírem o descritor “cluttering”, para taquifemia, não se obteve nenhum estudo voltado para o rastreamento e/ou avaliação do risco para a taquifemia em crianças pré-escolares. Logo, todos os resultados desta RE referem-se aos fatores de risco para a gagueira, bem como aos métodos avaliativos utilizados no rastreamento deste transtorno da fluência em pré-escolares. Acredita-se que esse resultado esteja associado com a falta de concordância em relação aos critérios diagnósticos da taquifemia, à escassez de evidências científicas consolidadas, e ao diagnóstico mais tardio deste transtorno^(39,40)^.

Contudo, observa-se que alguns fatores de risco identificados nesta RE mostram-se compatíveis com características clínicas observadas na taquifemia, dentre elas: sexo masculino^(11)^, alteração na velocidade de fala, prejuízo na inteligibilidade do discurso, incoordenação pneumofonoarticulatória^(12,18,25,35)^, presença de outras disfluências (repetição de palavra não monossilábica)^(12,25)^, e presença de comorbidades^(11,18,19,35)^. Uma vez que a taquifemia também é um transtorno da fluência que inicia na infância, questiona-se: será que a observância desses fatores de risco para a gagueira também não seria capaz de rastrear o risco para a taquifemia em pré-escolares? Se sim, é possível que alguns resultados obtidos desse rastreamento sinalizem um risco falso positivo para a gagueira.

Assim, crianças com risco para a taquifemia poderiam ser identificadas como crianças com risco para a gagueira. A identificação de fatores de risco comuns para gagueira e taquifemia, em métodos avaliativos exclusivos para o rastreamento da gagueira, aponta para a necessidade de atualização dos instrumentos formais para o rastreamento dos transtornos da fluência com início na infância. A exemplo de um estudo que se propôs em explorar a aplicabilidade do IRGD no contexto educacional, e concluiu que a aplicação do instrumento possibilitou a identificação de pré-escolares com risco para os transtornos da fluência, e não apenas para a gagueira^(41)^.

### O valor da queixa de gagueira referida pelos pais/responsáveis

A alta frequência de disfluências típicas da gagueira na fala da criança foi sinalizada como um importante fator de risco para a cronicidade da gagueira pela maioria dos estudos analisados^(12-14,19-21,25,35)^. Muitos pesquisadores e clínicos descrevem que a gagueira é caracterizada por prolongamentos, bloqueios e repetições da fala^(6,42)^, e que a porcentagem de disfluências típicas da gagueira ≥ 3% é um indicador de risco para gagueira persistente^(43)^. No entanto, a análise isolada desta característica não é adequada para concluir o diagnóstico, uma vez que determinadas populações, como crianças bilíngues, podem apresentar um diagnóstico falso positivo de gagueira^(44)^. Além disso, a gagueira apresenta uma alta variabilidade de suas manifestações, podendo apresentar valores diferentes de frequência das disfluências a depender dos interlocutores envolvidos em momentos e situações comunicativas distintas^(45)^.

A presença das disfluências típicas da gagueira na fala da criança ocasiona preocupação aos pais/responsáveis, que procuram o atendimento clínico com o intuito de confirmar o diagnóstico de gagueira. Em geral, os relatos dos pais/responsáveis são altamente precisos^(46)^. Neste sentido, a queixa referida pelos pais/responsáveis, pautada na observação da alta frequência de disfluências típicas da gagueira, consiste em uma ferramenta indispensável para a pesquisa e prática clínica^(45)^. De todos os fatores de risco mais preditivos para a gagueira, a queixa foi o mais citado nesta RE^(12,13,18-20)^, o que atribui o devido valor ao relato dos pais/responsáveis, e destaca a necessidade de acolhimento e escuta ativa das famílias cujas crianças possuem queixa de gagueira.

### Gagueira, sua base genética e a importância do fator tempo

Dentre os fatores de risco identificados nos onze estudos analisados, o histórico familial positivo para a gagueira foi o mais frequente^(11-14,18,19,21,25,35,36)^ e considerado um dos mais preditivos^(11,19,21)^. A gagueira é altamente hereditária, com variantes genéticas distintas compartilhadas entre os familiares que impactam o risco do transtorno^(6)^. Os modelos de transmissão genética da gagueira são consistentes com alta poligenicidade, sugerindo uma arquitetura genética complexa^(6)^, mas são os processos epigenéticos que influenciam a expressão gênica ao longo da vida^(47)^. Logo, os padrões de crescimento do sistema nervoso central e o fenótipo final (por exemplo, gagueira versus não gagueira) não são fixados no nascimento, mas surgem ao longo do desenvolvimento, por meio da interação de genes, processos epigenéticos e experiência^(36,47)^.

O tempo, por sua vez, é uma variável fundamental para a gagueira, que tem sido considerada no âmbito da Neurociência como um transtorno de timing neural^(48)^, pela presença de déficits na sincronia temporal necessária para a coordenação precisa dos impulsos elétricos e químicos no sistema nervoso central, fundamental para o processamento de informações. O início dos sintomas da gagueira ocorre no período do neurodesenvolvimento^(32)^, por isso, a necessidade de identificar o transtorno o mais próximo do surgimento dos seus sintomas, a fim de que a intervenção fonoaudiológica adequada seja precoce e com sucesso^(25,49,50)^. Quanto mais o tempo passa para uma criança que gagueja, mais aumenta a sua idade e a duração da gagueira, enquanto diminuem as suas chances de recuperação^(47)^, pois a persistência das disfluências típicas da gagueira por no mínimo doze meses foi um fator de risco prevalente para a maioria dos estudos analisados^(11-14,18,21,25,35)^.

### A reação dos outros em relação à fala da criança que gagueja importa?

Apesar de tanto a reação negativa da criança em relação à própria fala^(11-13,18,25,35,36)^, quanto à reação social negativa em relação à fala da criança (família estendida, escola e outros)^(12,13,18,25,35,36)^ terem sido identificadas na literatura revisada como fatores de risco para a gagueira, a reação social negativa foi a apontada como mais preditiva para a gagueira^(12,13)^. Tal constatação nos remete à necessidade de rastrear o risco para a gagueira a partir de uma perspectiva ampliada de cuidado, onde o domínio dos fatores contextuais é considerado tanto quanto o domínio das funções e estruturas do corpo, conforme a Classificação Internacional de Funcionalidade, Incapacidade e Saúde (CIF)^(51)^.

Identificar como fator de risco preditivo para a gagueira a reação social negativa da família, escola e outros em relação à fala da criança, é constatar a relevância do papel do ambiente sobre o período do neurodesenvolvimento, que sofre a influência da experiência^(36,47)^. Além de destacar a importância das práticas de educação em saúde, como ferramentas de transformação social capazes de reduzir o risco de desencadeamento ou manutenção da gagueira na população infantil. Dessa forma, é preciso que o cuidado com a criança que gagueja tenha uma perspectiva ampliada, para atender também ao domínio dos fatores contextuais, envolvendo por exemplo, orientações de promoção de fluência a famílias, escolas e outros setores sociais.

### Linguagem prejudicada é sinal de maior risco para a gagueira: por quê?

Dentre os fatores de risco analisados como os mais preditivos para a gagueira, observou-se: pior desempenho em avaliação articulatória/fonológica padronizada^(14,21)^; e desenvolvimento da linguagem abaixo da média^(14)^. Mas, por qual motivo a presença de tais alterações linguísticas aumentariam o risco de um pré-escolar gaguejar? Uma possibilidade explicativa é assumir a gagueira como um transtorno da fluência que inicia na infância^(32)^, e a fluência como uma habilidade linguística^(52)^. Conforme a Teoria Integrada da Fluência (TIF), fluência é uma habilidade da competência linguística, ou seja, ela faz parte do conjunto de capacidades e habilidades mentais que fazem toda e qualquer pessoa ser capaz de produzir e compreender a linguagem^(52)^.

Tanto crianças quanto adultos com gagueira apresentam dificuldades sensório-perceptuais que refletem padrões atípicos de desenvolvimento, seja na produção ou na compreensão da linguagem^(53,54)^. A presença de alterações estruturais na via dorsal do circuito da linguagem em adultos que gaguejam, também fornece suporte para conceber que a gagueira está relacionada com um comprometimento no mapeamento bidirecional entre os córtices auditivos e articulatórios suportados pelas vias dorsais^(55)^. A via dorsal é fundamental para a organização das palavras em sequência e para a geração de sentenças^(56)^, bem como é especialista no processamento perceptual e fonológico da linguagem falada, mediante a análise da complexidade acústica, desambiguação dos sons e integração funcional com o processamento auditivo^(57)^.

Diante desse cenário, já que a aquisição da linguagem transita de uma análise prosódica e distribucional do material da fala para uma análise sintática dos enunciados linguísticos, conforme a hipótese do *bootstrapping* fonológico^(58)^, a TIF lança as hipóteses de que: por ser a fluência uma habilidade linguística, seu desenvolvimento típico está diretamente relacionado aos processos ótimos de identificação e acesso a traços formais^^1^^ , enquanto a gagueira, por ser um transtorno da fluência, está relacionada a dificuldades na representação de traços formais, principalmente o traço categorial, no léxico mental^^2^^ e/ou em acessá-los durante a computação on-line^(52)^. Dificuldades nesses requisitos aquisicionais para desenvolvimento da linguagem podem afetar a compreensão e a produção da linguagem, além de ocasionar problemas na inicialização do sistema computacional^^3^^ da criança, prejudicando a implementação da sintaxe e o processo de identificação categorial das palavras, como o desenvolvimento típico necessita^(60)^.

A proposta conceitual sobre o que é fluência e hipóteses lançadas pela TIF foram verificadas experimentalmente, e a teoria se mostrou testável e com potencial para elevado poder explicativo, pois os achados confirmaram as hipóteses lançadas no estudo^(52)^. Então, a observação obtida nesta RE, de que fatores de risco relacionados à prejuízos no desenvolvimento da linguagem se mostram mais preditivos para a gagueira^(14,21)^, mostra-se convergente com a assunção teórica da fluência como uma habilidade linguística^(52)^. Fato este que deve sensibilizar a comunidade fonoaudiológica para implementar à sua prática mecanismos de avaliação da linguagem junto aos pré-escolares com queixa de gagueira, tanto no tocante à compreensão quanto à produção da linguagem. Portanto, as ações de rastreamento do risco para a gagueira envolvem também considerar a investigação de alterações da linguagem no desenvolvimento infantil.

### A escassez de instrumentos formais para rastreamento da gagueira e o protagonismo do Brasil

Este estudo identificou uma lacuna científica significativa, a escassez de instrumentos de rastreamento dos fatores de risco para os transtornos da fluência em pré-escolares. Dentre os métodos padronizados mapeados, apenas dois instrumentos foram identificados: o PRGD^(18,35)^ e o IRGD^(12,25)^. Destaca-se que ambos os instrumentos foram desenvolvidos por pesquisadoras brasileiras, colocando o Brasil como um país empenhado cientificamente em rastrear precocemente a gagueira. Isto possivelmente se deve ao fato de o sistema de saúde pública implementado no país possuir uma política nacional de promoção da saúde, com vistas a construir ações que possibilitam responder às necessidades sociais em saúde^(61)^. Esta política estimula o desenvolvimento de ações de promoção e prevenção à saúde em todos os níveis de atenção, dando ênfase, principalmente, na atenção primária^(61)^.

No Brasil, os profissionais da saúde que atuam na atenção primária à saúde, e os educadores que atuam na educação infantil, são os responsáveis por monitorar o desenvolvimento infantil e identificar os primeiros sintomas de alteração na saúde da criança^(62,63)^. Encontra-se em tramitação no país um projeto de lei^(64)^ que reconhece a necessidade de garantir às crianças que gaguejam o direito ao diagnóstico e tratamento precoce. Sendo assim, os instrumentos de rastreamento para serem aplicados no Brasil precisam ser de uso multiprofissional, visto que o cuidado à saúde na infância é descentralizado. Neste sentido, os achados desta RE identificaram que apenas o IRGD demonstrou possuir essa característica.

Os achados mostraram ainda que houve a combinação de vários métodos avaliativos utilizados na identificação dos fatores de risco e no diagnóstico da gagueira nos estudos analisados. Esta informação corrobora com descrições prévias de que a gagueira é um transtorno multifatorial, cuja avaliação multidimensional é a mais apropriada, a fim de determinar os fatores que podem estar contribuindo para a instabilidade observada na fluência da criança^(21,36,47)^.

### Um fator de risco isolado não é o suficiente

Pode-se considerar que há uma tendência de analisar os fatores de risco em associação, ou seja, a análise de múltiplos domínios é preconizada na avaliação do risco de uma criança desenvolver a gagueira persistente, uma vez que este apontamento foi realizado pela maioria dos estudos^(11,13,14,19-21)^. Analisar um fator de risco isoladamente não é considerado um indicador preditivo para gagueira, pois as crianças podem apresentar diferentes perfis de fatores de risco^(20,21)^. Estudos apontaram que, isoladamente, a queixa e o grau de severidade, por exemplo, não foram preditivos de eventual persistência ou recuperação em pré-escolares^(20,21)^. Quando a análise foi realizada considerando múltiplos fatores, a presença de pelo menos dois fatores preditivos mostrou-se significativamente associada a um risco elevado de desenvolvimento da gagueira persistente^(14,21)^.

Os fatores de risco identificados podem ser causais, desencadeantes ou mantenedores da gagueira, cuja análise preditiva de risco em crianças pré-escolares deve ocorrer de forma cumulativa, ou seja, recomenda-se considerar múltiplos fatores de risco^(14,19,20)^, e não apenas de um fator isoladamente. Especialistas recomendam que quanto maior o número de fatores de risco identificados, mais precoce deve ser o encaminhamento da criança para o cuidado especializado^(11,14)^. Logo, os instrumentos com finalidade de rastreamento para a gagueira devem ser elaborados numa perspectiva multifatorial, cujos itens devam compor múltiplos domínios, para que a análise de risco seja cumulativa e personalizada ao perfil de cada criança.

### Presente e futuro do rastreamento dos transtornos da fluência

O cenário atual do rastreamento dos transtornos da fluência é marcado por duas lacunas significativas: a ausência de estudos que investiguem fatores de risco para a taquifemia em pré-escolares e a escassez de instrumentos padronizados para rastreamento da gagueira nessa população, com métodos avaliativos ainda predominantemente dependentes da aplicação exclusiva por fonoaudiólogos. Em contrapartida, observa-se uma ampla diversidade de fatores de risco descritos na literatura, os quais revelam avanços significativos ao integrarem, além dos fatores biológicos, variáveis contextuais de relevância clínica, como fatores pessoais (inerentes à criança) e ambientais (inerentes à família estendida, escola e outros).

As perspectivas para estudos futuros devem priorizar o desenvolvimento e validação de instrumentos de rastreamento para os transtornos da fluência, mediante a inclusão da taquifemia como transtorno-alvo direto na investigação precoce, a ampliação e diversificação das amostras e a incorporação de tecnologias digitais que viabilizem o rastreamento precoce em larga escala e por diferentes profissionais. Esses avanços são fundamentais para aprimorar os métodos avaliativos utilizados, conferindo maior precisão na rastreabilidade dos transtornos da fluência na primeira infância. Além de favorecer tanto a identificação precoce quanto o acesso oportuno das crianças com transtornos da fluência à assistência em saúde.

### Limitações, recomendações práticas e implicações clínicas desta revisão de escopo

Esta revisão de escopo apresentou limitações relacionadas ao número reduzido de bases de dados consultadas e à heterogeneidade das amostras dos estudos, o que dificultou a comparação dos resultados. Houve também concentração de publicações oriundas de determinados grupos de pesquisas, resultando em metodologias repetidas e fatores de risco semelhantes. A predominância de estudos sobre gagueira, com escassa investigação da taquifemia, limita a generalização dos achados e reforça a necessidade de ampliar as evidências sobre os transtornos da fluência na primeira infância.

Apesar das limitações, a revisão traz contribuições importantes ao sistematizar dados que podem orientar o desenvolvimento e a validação de instrumentos de rastreamento dos transtornos da fluência na infância. Destacam-se os principais fatores de risco associados à persistência da gagueira e a análise crítica dos métodos avaliativos, oferecendo subsídios práticos para a identificação precoce e o acompanhamento clínico de crianças em idade pré-escolar.

Os achados desta revisão reforçam que o rastreamento precoce da gagueira deve considerar diversos fatores de risco combinados — como queixa dos pais, histórico familial, alta frequência de disfluências típicas da gagueira, o tempo de duração e a idade da criança no início do quadro — que servem como sinais de alerta para encaminhamento à avaliação fonoaudiológica. A adoção de protocolos de triagem multiprofissionais pode ampliar o alcance do rastreamento e favorecer intervenções mais precoces e efetivas.

Recomenda-se a atualização e validação dos instrumentos avaliativos para diferentes contextos socioculturais, incorporando variáveis relacionadas à taquifemia e à CIF. Sugere-se também o uso de tecnologias digitais para ampliar a detecção precoce e o desenvolvimento de pesquisas que considerem a diversidade dos fatores de risco e da população, fortalecendo a base empírica existente.

Os resultados desta pesquisa têm aplicação direta nas práticas de saúde e educação, oferecendo subsídios para políticas públicas e ações preventivas voltadas à promoção da fluência infantil. O rastreamento baseado em múltiplos fatores de risco e contexto familiar favorece estratégias de prevenção e oportuniza o tempo para intervenções precoces e eficazes.

## CONCLUSÃO

Esta revisão de escopo identificou evidências voltadas aos fatores de risco e métodos avaliativos da gagueira em pré-escolares, sem estudos específicos sobre taquifemia. Os principais fatores de risco incluíram queixa dos pais, histórico familial, alta frequência de disfluências típicas, tempo de duração e idade de início da gagueira. Verificou-se escassez de instrumentos padronizados e validados internacionalmente, com apenas dois protocolos brasileiros formais (PRGD e IRGD). Destaca-se a necessidade de desenvolver métodos acessíveis a diferentes profissionais e contextos, além de ampliar o rastreamento precoce. Constatou-se uma lacuna científica sobre a taquifemia, recomendando-se estudos que abordem seus fatores e métodos de avaliação na infância.

## Footnotes

1 Traços são os valores e as informações que se encontram codificadas no léxico de uma língua, de modo que cada item lexical consiste em um composto de traços. Traços formais orientam o sistema computacional a respeito das relações sintáticas que um dado item lexical deve estabelecer com outros no interior da sentença em que venha a ser inserido. Uma das atribuições do traço formal categorial é determinar a posição distribucional de um item na sentença^(59)^.

2 Léxico mental, aqui, é concebido como um repositório de conhecimentos de natureza fonológica, semântica, pragmática e estilística sobre os itens linguísticos^(59)^.

3 Sistema computacional também conhecido como gramática mental ou sintaxe é encarregada por retirar as informações do léxico mental para construir derivacionalmente representações sintáticas, por meio de operações computacionais^(59)^.
